# Mechanism for DPY30 and ASH2L intrinsically disordered regions to modulate the MLL/SET1 activity on chromatin

**DOI:** 10.1038/s41467-021-23268-9

**Published:** 2021-05-19

**Authors:** Young-Tae Lee, Alex Ayoub, Sang-Ho Park, Liang Sha, Jing Xu, Fengbiao Mao, Wei Zheng, Yang Zhang, Uhn-Soo Cho, Yali Dou

**Affiliations:** 1 Department of Pathology, University of Michigan, Ann Arbor, MI USA; 2 Department of Biological Chemistry, University of Michigan, Ann Arbor, MI USA; 3 Department of Medicine, Department of Biochemistry and Molecular Medicine, University of Southern California, Los Angeles, CA USA; 4 Computational Medicine and Bioinformatics, University of Michigan, Ann Arbor, MI USA; 5 Present Address: Accent Therapeutics, 65 Hayden Avenue, Lexington, MA USA

**Keywords:** Biochemistry, Enzyme mechanisms, Structural biology, Electron microscopy, NMR spectroscopy

## Abstract

Recent cryo-EM structures show the highly dynamic nature of the MLL1-NCP (nucleosome core particle) interaction. Functional implication and regulation of such dynamics remain unclear. Here we show that DPY30 and the intrinsically disordered regions (IDRs) of ASH2L work together in restricting the rotational dynamics of the MLL1 complex on the NCP. We show that DPY30 binding to ASH2L leads to stabilization and integration of ASH2L IDRs into the MLL1 complex and establishes new ASH2L-NCP contacts. The significance of ASH2L-DPY30 interactions is demonstrated by requirement of both ASH2L IDRs and DPY30 for dramatic increase of processivity and activity of the MLL1 complex. This DPY30 and ASH2L-IDR dependent regulation is NCP-specific and applies to all members of the MLL/SET1 family of enzymes. We further show that DPY30 is causal for de novo establishment of H3K4me3 in ESCs. Our study provides a paradigm of how H3K4me3 is regulated on chromatin and how H3K4me3 heterogeneity can be modulated by ASH2L IDR interacting proteins.

## Abstract (web summary)

Regulation of the MLL family of histone H3K4 methyltransferases on the nucleosome core particle (NCP) remains largely unknown. Here the authors show that intrinsically disordered regions of ASH2L and DPY30 restrict the rotational dynamics of MLL1 on the NCP, allowing more efficient enzyme-substrate engagement and higher H3K4 trimethylation activity.

## Introduction

Cells are complex, information-processing centers that handle an immense flow of signals often leading to fine tuning the expression of genes. To achieve exquisite regulation, chromatin post-translational modifications (PTMs) have evolved to demarcate, among a mosaic of functions, actively transcribed genes from the inactive ones^1^. The mixed-lineage leukemia (MLL) family of histone methyltransferases (HMTs) catalyzes the deposition of histone H3 lysine 4 methylation (H3K4me) associated with active transcription^2,3^. H3K4 methylation is highly enriched at gene promoters and distal regulatory enhancers, and plays a pivotal role in the recruitment of basal transcription machinery^4–6^ and chromatin remodeling complexes^7–9^. It also promotes long-range chromatin interactions and higher-order chromatin organization^10–12^. The dynamic interplay between H3K4me and co-transcriptional processes has also been reported^13,14^. Human genetic studies have corroborated the functional importance of the MLL family enzymes: heterozygous mutations in MLLs are reported in congenital human Kabuki^15–20^, Wiedemann-Steiner, and Kleefstra spectrum syndromes^21–23^. Furthermore, MLL family proteins are among the most frequently mutated genes in human malignancies^24^.

The MLL/SET1 family enzymes interact with several evolutionarily conserved proteins, WDR5, ASH2L, RbBP5, and DPY30, through the C-terminal catalytic SET domain^25–27^. We and others have previously shown that these core components are essential for MLL1 catalytic activity on histone H3^27–29^. In particular, WDR5 is required to stabilize the trimeric RbBP5-ASH2L-MLL1 complex^30,31^, a role exploited for the development of MLL1-specific inhibitors^32,33^. In-depth biochemical studies also show that these core components have multiple relatively weak interactions amongst themselves^34–36^. Recently, a co-crystal structure of the minimal trimeric complex (ASH2L^276–500,Δ400–440^-RbBP5^330–375^-MLL1/3^SET^)^30^ and cryo-EM structures of the MLL1-NCP (nucleosome core particle) complex^37,38^ have revealed the overall architecture of the MLL1 core complex as well as its engagement with a physiological substrate (i.e., NCP). These studies, together with solution structures of MLL1^35^, show a surprisingly dynamic nature of the MLL1 core complex, especially the MLL1^SET^ domain and the RbBP5-NCP interface. Despite these studies, regulation of structural dynamics of the MLL1 complex on the NCP and its functional implication remain largely unknown.

Compared to the well-studied WDR5, RbBP5, and ASH2L^SPRY^ proteins, the function of DPY30 and the extended intrinsically disordered regions (IDRs) of ASH2L in the MLL1 complex remains a mystery. The biochemically defined minimal core complex showed negligible DPY30 contribution to the activity of the MLL/SET1 family of enzymes using recombinant histone H3 or peptidic H3 as substrates^36,39,40^. On the other hand, DPY30 is capable of regulating global H3K4 methylation in cells^41^ and DPY30 knockdown or knockout leads to global reduction of H3K4me3 in embryonic stem cells (ESCs) and hematopoietic stem cells^42,43^. It is proposed as a potential therapeutic target for MLL1-rearranged leukemia^44^. The conflicting reports of the minimal in vitro DPY30 activity versus its importance in regulating H3K4me3 in cells remain unresolved.

Here we show that DPY30 greatly stimulates MLL1 activity on the NCP. By combined NMR, SAXS, cryo-EM and biochemical approaches, we find that DPY30 functions through the extended IDRs of ASH2L to restrict the rotational dynamics of the MLL1 complex on the NCP, thereby promoting H3K4 methylation at higher methylation states. The NCP-specific regulation by DPY30 and ASH2L IDRs generally applies to all MLL/SET1 family enzymes. Cellular studies further confirm the importance of DPY30 in de novo establishment of H3K4me3 on chromatin. Taken together, we have established a paradigm of how the disordered regions in the chromatin-modifying complexes may exert loci-specific histone methylation and confer heterogeneity in the cellular epigenetic landscape.

## Results

### Activity of the MLL/SET1 family enzymes on the NCP requires DPY30

**Fig. 1:**
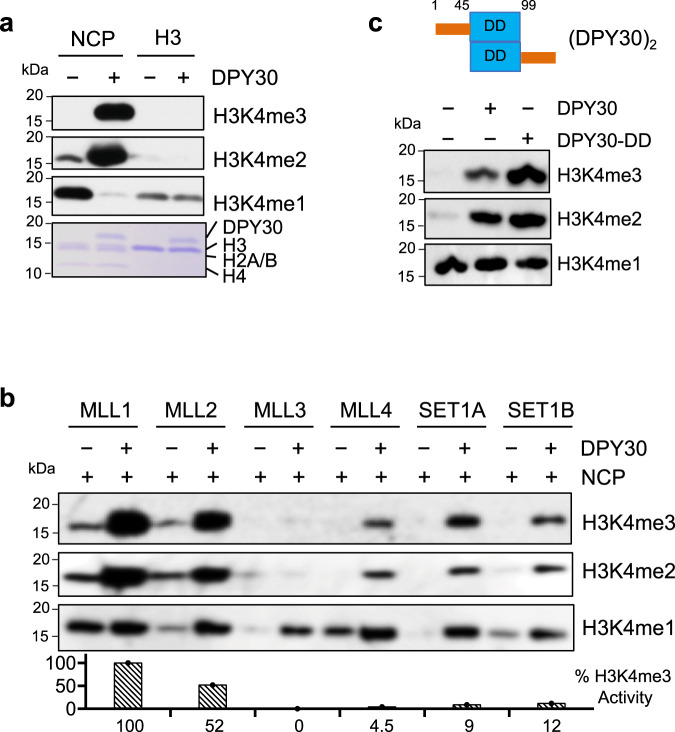
DPY30 specifically stimulates MLL1 activity on the NCP. **a** In vitro HMT assay for the MLL1 core complex using either the NCP (nucleosome core particle) or recombinant histone H3 as substrates, which were indicated on top. The MLL1 core complex (i.e., MLL1^SET^, WDR5, RbBP5, and ASH2L) was added with or without DPY30. Histones were run on 15% SDS-PAGE and blotted with anti-H3K4me1, H3K4me2, and H3K4me3 antibodies as indicated at right. The Coomassie gel was included as the loading control at bottom. **b** In vitro HMT assay for the core complexes of the MLL/SET1 family methyltransferases using the NCP as the substrate. The MLL/SET1 core complexes were added with or without DPY30 as indicated on top. **c** Top, Domain structure for the DPY30 dimers. DD, dimerization domain (blue). Bottom, in vitro HMT assay for the MLL1 core complex with no, dimerization domain only, or full-length DPY30. The NCP was used as the substrate in all reactions. Quantification completed using ImageJ^108^ with %activity calculated relative to wild-type ASH2L-containing complex^108^.

To examine the regulation of the MLL1 methyltransferase activity on the NCP in vitro, we performed the HMT assays using either recombinant histone H3 or NCP as substrates. The overall activity of the MLL1 core complex was much higher on the NCP as compared to recombinant histone H3 (Fig. 1a and Supplementary Fig. 1a). DPY30 was essential for the drastic increase of H3K4 methylation on the NCP (Fig. 1a), especially for higher H3K4 methylation states (i.e., H3K4me2 and H3K4me3) at the expense of H3K4me1 (Fig. 1a). In contrast, DPY30 had no effect on MLL1 activity or processivity when recombinant H3 was used as the substrate (Fig. 1a and Supplementary Fig. 1b), consistent with the previous studies^36,39,40^. To test whether DPY30-dependent regulation on the NCP is a general mechanism for all MLL/SET1 family enzymes, we examined H3K4 methylation by MLL2-4 and SET1A/1B in the presence or absence of DPY30. As shown in Fig. 1b and Supplementary Fig. 1b, DPY30 was able to significantly enhance methylation activity of all MLL/SET1 complexes in an NCP-specific manner. Domain mapping confirmed that the dimerization domain (DD, 45–90) of DPY30, which forms a hydrophobic groove that directly interacts with the ASH2L Sdc-DPY30-Interacting domain (SDI, 504–534)^45^, was sufficient to stimulate MLL1 activity on the NCP (Fig. 1c).

### DPY30-dependent stimulation requires IDR in ASH2L

The recent cryo-EM studies of the MLL1/3-NCP complexes show that DPY30 does not make direct contact with the NCP^37,38^. Consistently, when we tested the binding of the MLL1 complex to the NCP with or without DPY30, DPY30 did not alter MLL1-NCP interaction in a gel mobility shift assay (Supplementary Fig. 1c). We next tested whether DPY30-mediated stimulation is redundant with that of H2BK120 ubiquitylation (H2BK120ub), which enhances H3K4 methylation without altering the binding affinity of the ySET1 complex to the NCP^46^. As shown in Supplementary Fig. 1d, DPY30 was able to further enhance activities of SET1A and MLL1 on the H2BK120ub-containing NCP, suggesting that it functions through a distinct mechanism from that of H2BK120ub.

**Fig. 2:**
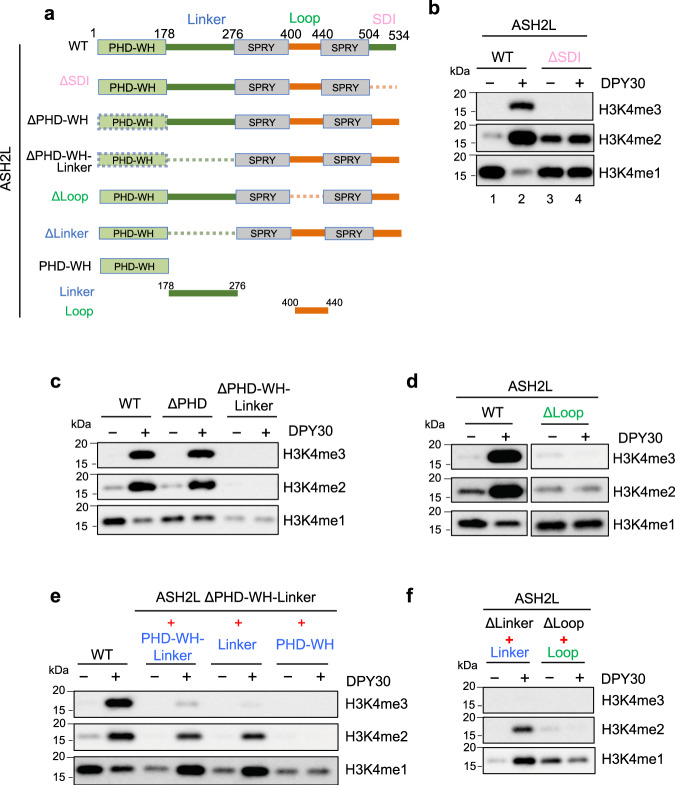
DPY30 requires ASH2L IDRs to stimulate MLL1 activity on chromatin. **a** Human ASH2L truncation and deletion mutants used in the in vitro HMT experiments. ASH2L contains two structural domains, the PHD-WH (plant homeotic-winged helix) domain on the N-terminus and a split-SPRY domain on the C-terminus. It also contains three IDRs, Linker (179-275), Loop (401–439), and SDI (504–534). **b** In vitro HMT assay for the MLL1 core complex with either wild type or ΔSDI ASH2L. **c** In vitro HMT assay for the MLL1 core complex with wild-type or ASH2L mutants as indicated on top. **d** In vitro HMT assay for the MLL1 core complex with wild type or ΔLoop ASH2L. **e** In vitro HMT assay for the MLL1 core complex with either wild-type ASH2L or a mixture of two stoichiometric ASH2L fragments as indicated on top. **f** Test the transactivation capability of Linker and Loop IDRs. In vitro HMT assay for the MLL1 core complex containing a mixture of Linker and ASH2L ΔLinker polypeptides or Loop and ASH2L ΔLoop polypeptides as indicated on top. For (**b**–**f**), in vitro HMT assays were performed with or without DPY30. An equal amount of the NCP was used in each reaction and histone methylation was detected by immunoblot using antibodies as indicated on right.

**Fig. 3:**
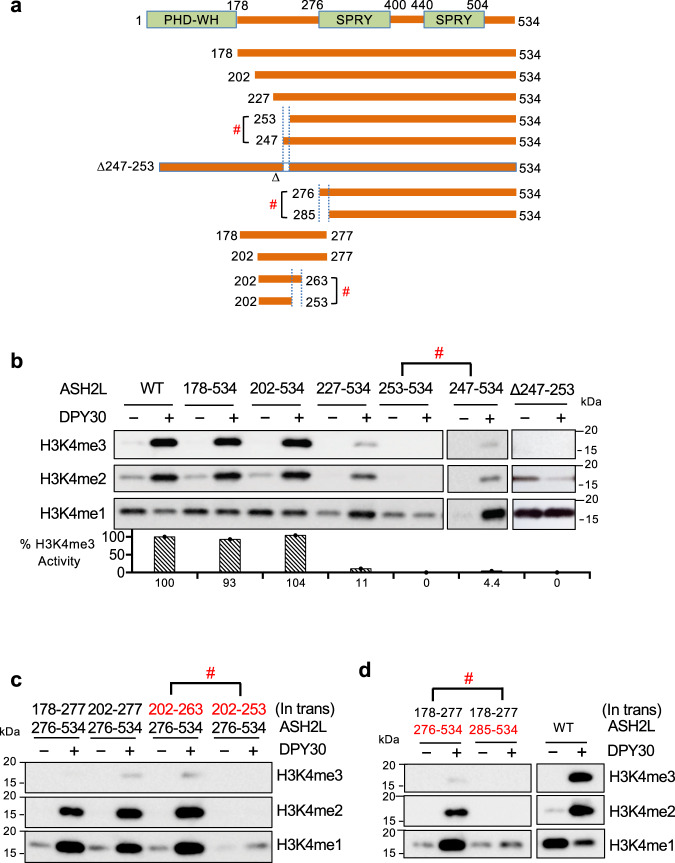
Identification of essential ASH2L IDRs in DPY30-mediated stimulation. **a** Human ASH2L deletion mutants used in (**b**–**d**) and transactivation peptides used in (**c**) and (**d**)**. b** Serial deletion to map essential ASH2L Linker IDRs for DPY30 function. In vitro HMT assay for the MLL1 core complex containing wild-type or various ASH2L mutants as indicated on top. The assays were performed in the presence or absence of DPY30. **c**–**d** Trans-activation experiments using two fragments of ASH2L in the in vitro HMT assay. The MLL1 core complexes containing a mixture of two stoichiometric ASH2L fragments were used with or without DPY30 as indicated on top. #, indicates abolishment of DPY30-dependent activity. Quantification completed using ImageJ^108^ with %activity calculated relative to wild-type ASH2L-containing complex.

As ASH2L is only the direct binding partner of DPY30 in the MLL1 core complex, we examined the role of ASH2L in DPY30-dependent regulation. ASH2L contains the structurally defined N-terminal PHD/WH domain (aa 1–178)^47,48^ and C-terminal split SPRY domain^49^ as well as three IDRs (Fig. 2a), including Linker (aa 178–275), Loop (aa 400–440), and SDI (aa 504–534). The SDI of ASH2L directly interacts with DPY30^50,51^, while both Linker and Loop are IDRs have not been previously characterized. In fact, Loop IDR was removed in the previous structural studies, as it does not contribute to the structural integrity of ASH2L SPRY^30,52^. We made selective serial deletions for each of these domains or regions in ASH2L (Schematic in Fig. 2a) to test their respective contribution to DPY30-dependent stimulation in the in vitro HMT assays. As shown in Fig. 2b, while SDI deletion increased activity of the MLL1 complex, likely by reducing ASH2L aggregation through SDI dimerization^52^, it completely eliminated DPY30-dependent stimulation on the NCP (Fig. 2b, lane 2 versus lane 4). Deletion of PHD-WH-Linker or Loop, but not PHD-WH alone, also abolished DPY30-dependent regulation (Fig. 2c, d). Interestingly, both PHD-WH-Linker and Linker fragments were able to stimulate MLL1 activity in a DPY30-dependent manner in trans, albeit at a lower level compared to *cis*-regulation (Fig. 2e, f). This property was not shared by Loop IDR in the HMT assay (Fig. 2f). Furthermore, detailed mapping of ASH2L Linker IDRs (Fig. 3a) identified three highly conserved regions, 247–251, 252–263 and 275–285 (Supplementary Fig. 2a), that were critical for DPY30-dependent regulation (Fig. 3b–d). These results highlight a previously uncharacterized function of ASH2L IDRs in regulating MLL1 activity on the NCP.

We next examined whether IDRs in other complex subunits are important for DPY30-mediated HMT stimulation. The potential IDRs in the MLL1 core complex include the RbBP5 C-terminus (aa. 382–538) and a segment of the SET domain between the WIN motif and the catalytic domain (aa. 3767–3812)^30^. Sequential C-terminal RbBP5 truncations were tested and none of them abolished DPY30-mediated HMT stimulation (Supplementary Fig. 2b). Notably, larger deletion of RbBP5 C-terminus lowered the overall HMT activities (Supplementary Fig. 2b), consistent with previous studies for yeast homolog Swd1 in the SET1 complex^53,54^. To test the MLL1 SET IDR, MLL^SETIL (3813–3969)^ was used so that the MLL1 complex remains active in the absence of the WIN motif or WDR5^30^. Removal of the SET IDR did not affect DPY30-dependent stimulation (Supplementary Fig. 2b). Circumvention of WDR5 in the MLL1^SETIL (3813–3969)^-containing core complex also indicates that WDR5 is dispensable for DPY30-mediated stimulation. These results suggest that ASH2L IDRs are necessary and sufficient for DPY30-dependent HMT stimulation on the NCP.

### DPY30 induces widespread NMR spectra changes in ASH2L IDRs

**Fig. 4:**
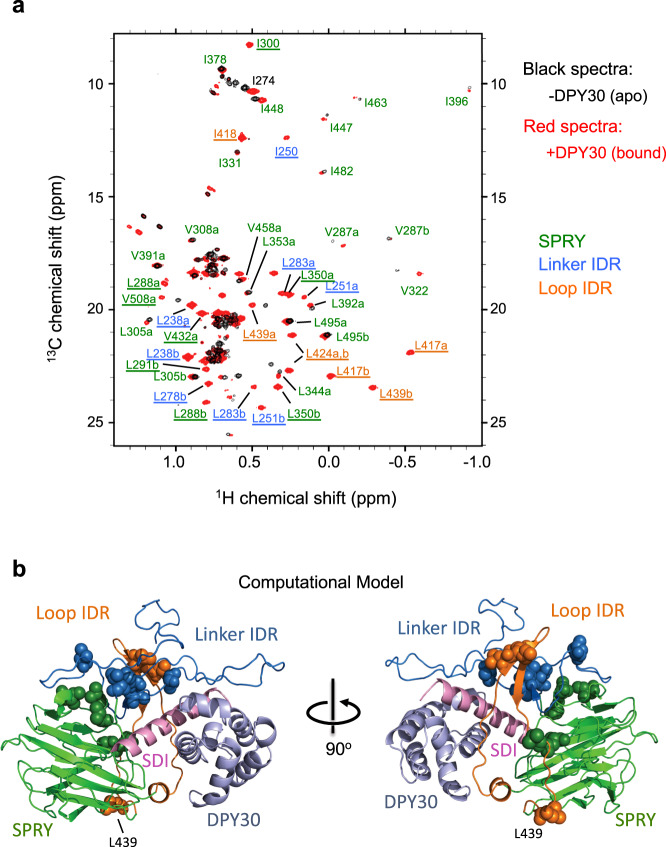
ASH2L IDRs undergo significant conformational changes upon DPY30 binding. **a** DPY30 binding induces drastic conformational change in ASH2L. Superimposed methyl-TROSY spectra of [^2^H, ^13^CH_3_-ILV] ASH2L^202–534^ in the absence (black) or presence (red) of DPY30. The labels indicate assigned residues in the DPY30-bound state. Underlined residues are newly appeared peaks upon DPY30 addition. **b** Computation model for ASH2L IDRs after DPY30 binding. Underlined residues in (**a**). are presented as spheres. These residues clustered together into a compact structure in this model. For both **a** and **b**, SPRY domain is shown in green, Linker IDR is shown in blue, Loop IDR is shown in orange.

To evaluate the effects of DPY30 binding on global ASH2L structure and to explore the mechanism by which DPY30 and ASH2L IDRs regulate MLL1 activity, we performed methyl-TROSY NMR on ^13^CH_3_-labeled Ile-Leu-Val (ILV) ASH2L^202–534^, in the presence of stoichiometric amount of unlabeled RbBP5 peptide (330–363), the minimal region for ASH2L binding (see Methods for details). We identified ~65% of the 100 anticipated peaks in ^13^CH_3_-labeled ILV ASH2L^202–534^ (Supplementary Fig. 3a, red). The majority of these peaks were also observed in the ^13^CH_3_-labeled ILV ASH2L^276–534^ (i.e., without Linker) sample (Supplementary Fig. 3a, black). Surprisingly, the addition of DPY30 triggered striking and widespread changes in the NMR spectrum, with the appearance of many new peaks with significantly dispersed chemical shifts (Fig. 4a and Supplementary Fig. 3b and 3c, red peaks). Chemical shift changes of some apo-state peaks were also observed (Fig. 4a). To further characterize these newly appeared peaks, we carried out residue-specific methyl-assignments by mutagenesis on the ASH2L^202–534^-DPY30 complex (Supplementary Fig. 4a–d)^55^. About 60% of total methyl peaks were unambiguously assigned (see Supplementary Table 1), owing to their dispersed chemical shifts. Interestingly, the majority of the DPY30-induced new peaks corresponded to residues in the ASH2L Linker and Loop IDRs (Fig. 4a, blue and orange, respectively). A number of peaks corresponding to residues in the SPRY domain (Fig. 4a, green) were also perturbed (e.g., I274, V287, I300, V322, I488) or newly appeared (e.g., L291, L350). Importantly, deletion of either Linker (blue) or Loop (orange) IDRs in ASH2L (modeled in Supplementary Figs. 5 and 6) abolished DPY30-induced changes in NMR spectra (Supplementary Fig. 6a–c, right). The NMR results suggest that DPY30 mainly affects ASH2L IDRs and the DPY30-dependent NMR changes require all ASH2L IDRs.

### Small-angle X-ray scattering (SAXS) of ASH2L and ASH2L/DPY30

The DPY30-dependent changes of ASH2L IDRs in NMR spectra can be due to alterations of inter-or intra-molecular interactions or stabilization of a particular conformation. To gain more insights into these possibilities, we performed SAXS experiment for ASH2L, DPY30, and the ASH2L/DPY30 complex. The molecular weight for ASH2L was estimated to be 65 KDa by the SAXS experiment. Since the combined mass of ASH2L (60.12 KDa) and RbBP5^330–363^ (4.07 KDa), which was included in all ASH2L SAXS samples (see “Methods”), is ~64 kDa, ASH2L is likely monomeric in solution. This excludes the possibility that DPY30 functions through resolving intermolecular interactions of ASH2L IDRs. Furthermore, SAXS data show that pair distance distribution function of ASH2L had a peak around 30 Å and decreased smoothly (Supplementary Fig. 7a), suggesting that the structural domains in ASH2L were probably not locked in a rigid configuration. As shown in Supplementary Fig. 7a, ASH2L/DPY30 had a similar D_max_ (~140 Å) as compared to ASH2L despite a 30% increase in size (Supplementary Fig. 7a). It suggests that ASH2L in the DPY30/ASH2L complex is probably in a more compact conformation. Interestingly, analysis using ensemble-optimized method (EOM)^56^ identified two distinguishable ASH2L populations in both the *D*_max_ and *R*_g_ plots (Supplementary Fig. 7b), suggesting that ASH2L is likely in a structural equilibrium between two largely different conformations, with one more extended than the other (Supplementary Fig. 7b). We were not able to perform EOM analysis for ASH2L/DPY30 due to method limitation^56^. Taken together, we speculate that DPY30 binding may shift the structural equilibrium of ASH2L and stabilize ASH2L IDRs in a more compact conformation. This is consistent with the DPY30-dependent appearance of ASH2L NMR peaks with well-dispersed chemical shifts (Fig. 4a).

### Molecular modeling of the DPY30-ASH2L complex

While it is challenging to determine the exact conformation(s) of the dynamic ASH2L IDRs in the apo-state, we were able to build a structural model to visualize ASH2L IDRs in the DPY30-bound state. The molecular model of the human ASH2L-DPY30 is based on the co-crystal structure of the ySET1 complex subunits Bre2-Sdc1 (PDB code: 6CHG)^53^ as well as crystal structures of the human ASH2L SPRY domain (without Loop IDR, PDB code: 3TOJ)^47^ (Fig. 4b, see “Methods”). When we mapped the residues that showed DPY30-dependent chemical shift in the NMR spectra onto this structural model, the close spatial proximity of these residues was apparent (Fig. 4b). They clustered together in the IDRs (Supplementary Fig. 5a) and SPRY regions (Supplementary Fig. 5b). In this model, ASH2L IDRs, the SPRY domain, and SDI adopt a compact triangular structural arrangement upon interacting with DPY30 (Fig. 4b). ASH2L IDRs form an ordered three-strand β-sheet, comprised of highly conserved residues 247–252 from Linker IDR and residues 416–428 from Loop IDR (Supplementary Fig. 8a, red box). In addition to the β-sheet structure, residues 252–263 and 275–286 of the Linker IDR also adopt a β-sheet-like conformation next to SDI (Supplementary Fig. 8a, blue box), enclosing a binding interface for the α-helical SDI (orange) and DPY30 (Supplementary Fig. 8b). Although this is only a computational model, many highlighted structural elements are essential for DPY30-dependent stimulation in the in vitro HMT assays. Removal of residues 247–253 or 400–440 completely abolished DPY30-dependent MLL1 regulation in vitro (Fig. 3b, d). Similarly, deletion of residues 252–263 or 275–285 in ASH2L also reduced DPY30-dependent activity (Fig. 3c, d) as well as the DPY30-dependent changes in NMR spectrum (Supplementary Fig. 6a–c).

### DPY30/ASH2L IDRs restrict the rotational dynamics of the MLL1 complex on the NCP

**Fig. 5:**
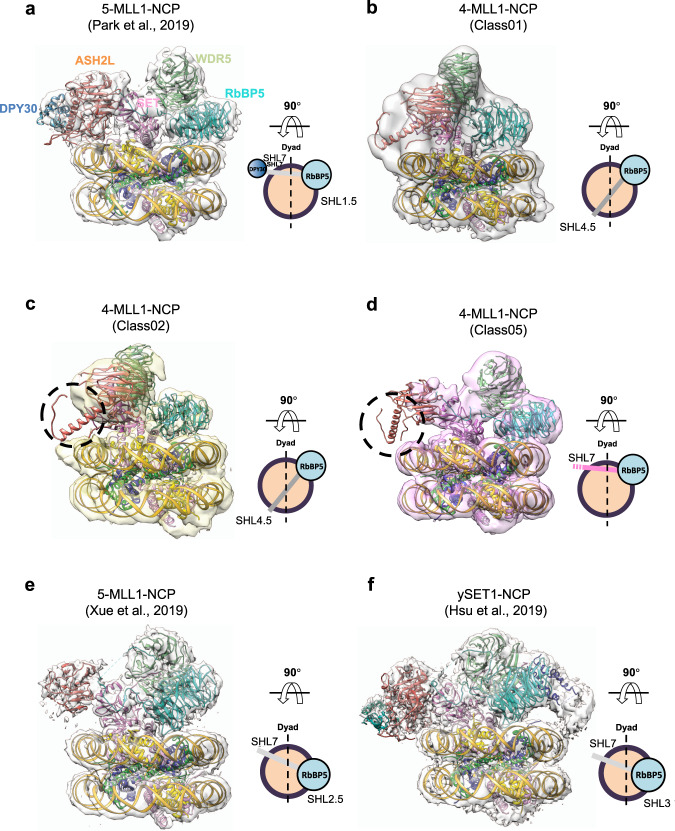
Cryo-EM structure of the 4-MLL1-NCP complexes. **a** Front views of the MLL1^RWSAD^-NCP (PDB ID: 6PWV [https://doi.org/10.2210/pdb6pwv/pdb] and EMDB: EMD-20512)^37^. The 90**°** top view, on right, shows the relative position of the anchoring points. DNA, yellow; DPY30, blue; ASH2L, orange; WDR5, light green; RbBP5, cyan. The NCP (histone octamer core, orange circle; DNA, black), RbBP5 (cyan circle), and DPY30 (blue circle), and the remaining MLL components were displayed as a gray bar indicating the orientation of the MLL1 complex. **b**–**d** Front view of the 4-MLL1-NCP structures. The 90**°** top view shows its anchoring points on the NCP. The missing EM density of ASH2L IDRs and DPY30 were indicated as black dash-circle in (**c**–**d**) and as a dashed line end in the accompanying cartoons. **e**–**f** Alternative conformation for the human 5-MLL1-NCP structure^38^ and ySET1-NCP^46^ as well as their respective top view. ASH2L/Bre2 in both structures interact with the NCP near SHL 7 with slight rotational dynamics at the RbBP5/Swd1-NCP contact points.

Recently, we and others have solved the cryo-EM structure of the MLL1-NCP complex^37,38^. It reveals the overall architecture of the five component MLL1 core complex with the NCP. In the MLL1-NCP structure, ASH2L binds to the NCP at DNA superhelical loop (SHL) 7 (Fig. 5a), which together with RbBP5 at SHL 1.5, allows MLL1^SET^ to bind above the nucleosome dyad^37^. To understand the molecular mechanism by which DPY30 regulates MLL1 activity on the NCP, we determined the single-particle cryo-EM structure of the human recombinant MLL1^RWSA^ complex (4-MLL1), containing four of the five core proteins, i.e. RbBP5 (aa 1–538); WDR5 (aa 22–334); MLL1^SET^ (aa 3762–3969); and ASH2L^ΔSDI^ (aa 1–504), bound to the NCP (4-MLL1-NCP). Overall, a total of 1288 K particles were picked from 6242 micrographs collected from 300 keV Titan Krios equipped with the K2 summit direct director (Supplementary Fig. 9). After several rounds of heterogeneous refinement using cryoSPARC^57^, we isolated four different subclasses of 4-MLL1-NCP (Class01, 02, 03, and 05). The best behaving particles were further selected from each subset of the 4-MLL1-NCP images after focused refinement and subsequent 3D classification in RELION (Supplementary Fig. 9)^58^. In the end, we obtained three different subclasses of 4-MLL1-NCP structures (Class 01, 02, and 05, Fig. 5b–d and Supplementary Fig. 9). The overall resolution of these structures ranged from 4.6 Å to 6.9 Å (Supplementary Fig. 10), which were sufficient to dock coordinates of the MLL1 core components and the NCP from our previous MLL1^RWSAD^-NCP structure (PDB ID: 6PWV [10.2210/pdb6pwv/pdb])^37^. In comparison to the MLL1^RWSAD^-NCP complex (or 5-MLL1-NCP, Fig. 5a)^37^, the 4-MLL1-NCP complexes displayed much higher dynamics at the ASH2L-NCP interface (Fig. 5b, c). While the majority of the 5-MLL1-NCP complexes anchored on the NCP with RbBP5 and ASH2L at DNA SHL 1.5 and 7, respectively, the 4-MLL1-NCP complex adopted multiple modes of interaction. With RbBP5 anchoring near SHL 1.5, ASH2L binding sites varied from SHL 7 to SHL 4.5 among different subclasses (Fig. 5b, d). Furthermore, local ASH2L binding dynamics on the NCP also increased significantly in the absence of DPY30, as demonstrated by extremely low or complete loss of ASH2L IDR density in a significant subset of the structures (Fig. 5c, d).

The molecular modeling using the iterative template-based fragment assembly refinement (I-TASSER) method^59,60^ showed that ASH2L IDRs make multiple contacts with nucleosomal DNA (Supplementary Fig. 11a). In addition to the conserved basic residues (_205_-KRK-_207_) that contributes to overall MLL1 activity on the NCP^37^, DPY30-induced ASH2L changes appear to enable ASH2L residues 419–421, which reside on a short loop between the newly formed three-stranded β-sheet, to provide another contact with DNA (Supplementary Fig. 11a). Consistent with the modeling, K419A/K421A mutation or deletion of 419–421 significantly reduced or abolished DPY30-dependent regulation of MLL1 activity, respectively (Supplementary Fig. 11b). These results are consistent with a model that DPY30 functions through ASH2L IDRs to restrict rotational dynamics of the MLL1 complex on the NCP and promote productive H3K4 methylation (see “Discussion”).

### DPY30 is essential for establishing de novo H3K4me3 in E14 ESCs

**Fig. 6:**
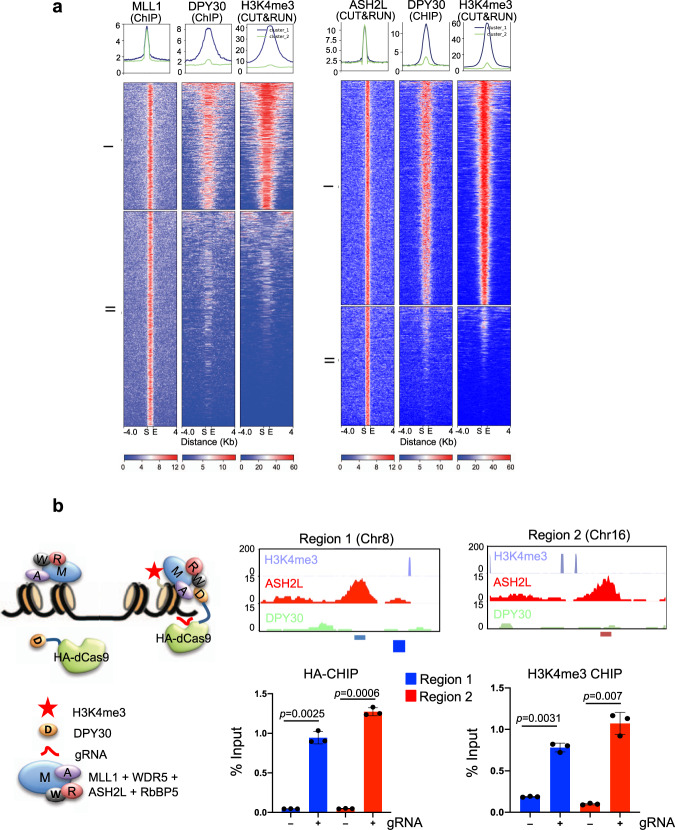
DPY30 regulates de novo establishment of H3K4me3 on chromatin. **a** DPY30 binding is highly correlated with H3K4me3 at the MLL1 binding sites. Heat map for 4009 MLL1 (left) and 2431 ASH2L (right) peaks and the corresponding DPY30 and H3K4me3 signals in ESCs. The signal as from merged biological duplicates. MLL1 or ASH2L peaks were clustered with *K*-means (*K* = 2) using normalized read counts at each peak. Two clusters were highlighted on left. Each row represents an 4 kb region up- and down-stream of the peak center. Peaks were sorted based on normalized read counts in each cluster. **b** DPY30 is able to establish de novo H3K4me3 on chromatin. Left, Experimental design for gRNA-mediated recruitment of dCas9-DPY30. The dCas9-DPY30 is recruited by gRNA to chromatin loci with prior binding of ASH2L and MLL1 and promotes H3K4me3 on chromatin. In the absence of gRNAs, dCas9-DPY30 is not recruited to target loci. W, WDR5; R, RbBP5; A, ASH2L; M, MLL1; D, DPY30. Right top, UCSC browser views of two randomly selected genomic regions are bound by ASH2L, but not DPY30. These regions do not have prior H3K4me3. Regions for gRNAs were highlighted on bottom. Right bottom, ChIP assay for HA-dCas9-DPY30 (left) or H3K4me3 (right) in cells transfected with or without the pooled gRNAs. ChIP signals were normalized against input and presented as %Input. Means and standard deviations (error bars) from at least three independent experiments were presented. Two-sided student *t* test was performed to calculate *p*-value.

To investigate the function of DPY30 in establishing H3K4me3 in cells, we first examined the correlation of DPY30 binding and H3K4me3 at MLL1 binding sites in E14 ESCs^41,60^. We identified 4009 MLL1 peaks in ESCs^61^ and among them, 1070 (26.69%) MLL1 peaks overlapped with those of DPY30 (Fig. 6a)^41^. Selected loci were shown in Supplementary Fig. 12a. Strikingly, H3K4me3 was highly correlated with DPY30 binding at the MLL1 targets (Fig. 6a). A similar close correlation of DPY30 and H3K4me3 was also found at the 2431 ASH2L binding sites, 67% of which colocalized with DPY30 at gene regulatory regions in the E14 ESCs (Fig. 6b and Supplementary Figs. 12b and 13). These results showed that MLL1/ASH2L alone was ineffective for depositing H3K4me3 on chromatin. Instead, DPY30 was required for promoting high levels of H3K4me3 on chromatin. Next, we tested whether DPY30 plays a causal role in establishing de novo H3K4me3 on chromatin. To this end, we expressed catalytically inactive HA-dCas9 or HA-dCas9-DPY30 in E14 cells and targeted the fusion proteins to randomly selected genomic regions by gRNAs (Fig. 6b, left). The loci were selected from MLL1/ASH2L joint targets that had no prior DPY30 binding (Fig. 6b, right top). Upon HA-dCas9-DPY30 recruitment, there was a significant increase of H3K4me3 at these loci (Fig. 6b, bottom right). In contrast, no increase of H3K4me3 was observed for the no gRNA controls (Fig. 6b) or in cells expressing HA-dCas9 (Supplementary Fig. 12c). These results confirmed that DPY30 is required for de novo establishment of H3K4me3 in cells.

## Discussion

Using the biochemical, structural, and cellular approaches, we have revealed the mechanism by which DPY30 regulates MLL//SET1 activity on chromatin. We show that DPY30 functions through ASH2L IDRs and DPY30-induced changes stabilize ASH2L-NCP interactions and restrict the rotational dynamics of the MLL1 complex on the NCP. Consequently, it promotes productive H3K4 methylation, especially at higher methylation states (i.e., H3K4me3 and H3K4me2). Our study has established a paradigm by which IDRs, the often-ignored segments in chromatin-interacting proteins, contribute to the heterogeneity of the epigenetic landscape in eukaryotic cells.

Previous studies have shown that DPY30 has negligible effects on H3 methylation in vitro^36,39,40^, yet its deletion leads to global downregulation of H3K4me3 in cells^41^. Our study shows that DPY30 confers NCP-specific regulation of MLL1 activity by regulating ASH2L-NCP interactions. Combining complementary biophysical, structural, and biochemical experiments with computational modeling, we show that upon DPY30 binding, ASH2L IDRs converge to adopt a compact structural unit at the MLL1-NCP interface, enabling new contacts with the NCP. In support, deletion or mutating ASH2L IDRs greatly impaired DPY30-dependent methyltransferase activity in vitro (Fig. 3 and Supplementary Fig. 11b). The cryo-EM structure of the 4-MLL1-NCP complex shows significant rotational dynamics on the NCP as compared to the 5-MLL1-NCP, 5-MLL3-NCP (Fig. 5e) or ySET1-NCP complexes (Fig. 5f)^37,38,46,62^. The 4-MLL1 complex is able to swing across the nucleosome disc with ASH2L binding near SHL4 in a subset of the cryo-EM structures (Fig. 5). Furthermore, ASH2L also exhibits higher local binding dynamics in the absence of DPY30. We envision that increased rotational dynamics of the 4-MLL1 complex or local ASH2L dynamics reduces the probability of the MLL1 SET domain positioning near nucleosome dyad. In this scenario, the MLL1^SET^ domain has to go through multiple spatial arrangements to optimally engage both H3 substrates in the NCP, which negatively affect MLL1 processivity^37^. By limiting rotational dynamics of the MLL1 complex on the NCP, DPY30 as well as ASH2L IDRs promote productive enzyme-substrate engagement, which has a specific impact on higher methylation states.

Notably, DPY30/ASH2L IDRs regulate all MLL/SET1 family enzymes, regardless of their respective intrinsic activity and processivity (Fig. 1b). We find that despite its selective impact on global H3K4me3 in cells^41^, DPY30 is able to stimulate H3K4me1 by the MLL3 complex in vitro. The global reduction of H3K4me3, but not H3K4me1 or H3K4me2, after DPY30 deletion/depletion in cells is probably due to compounding effects of relative abundance and activity of different MLL family enzymes as well as the offset of H3K4me1 inhibition by blocking its conversion to higher methylation states. We also would like to point out that DPY30 is able to enhance human SET1 activity on the H2BK120ub-containing NCP (Supplementary Fig. 1d). Thus, it can probably cooperate with H2BK120ub in H3K4me3 regulation in vivo, which awaits future studies.

It is well established that intrinsically disordered proteins (IDPs), or proteins containing extensive IDRs, have unique biophysical properties^63,64^. The undefined structures in the solution enable IDRs to adopt many possible conformations and meaningfully engage in versatile protein-protein interactions^65–67^. As a result, IDRs or IDPs are often found at hubs of protein interaction networks and enable functional diversification and environmental responsiveness during the complex developmental processes^66,68^. Recent studies also show that IDRs are able to facilitate phase transition and heterochromatin functions in cells^69^. Our study here provides a paradigm for how IDRs in histone-modifying enzymes may regulate chromatin functions. We show that ASH2L IDRs and their interacting protein DPY30 can exert locus- and context-specific regulation of H3K4me3 in cells. While the exact conformation(s) of apo-state ASH2L IDRs remain to be determined, our study suggests that ASH2L IDRs are probably in a highly dynamic conformational equilibrium and DPY30 binding leads to stabilization of ASH2L IDRs in one of the more structurally organized conformations. Our study also raises the question of whether ASH2L IDRs can be modulated by other proteins beyond DPY30. We envision that proteins that are able to induce perturbations in ASH2L IDRs and/or stabilize ASH2L IDRs could potentially modulate MLL/SET1-NCP interactions, thereby regulating H3K4 methylation activity on chromatin. Aberrant expression of ASH2L has been reported in a wide spectrum of human tumors, and contributes to disease progression and prognosis^24,70–72^. Notably, ASH2L cooperates with activating mutations of Ras in cellular transformation^73^, recruits the oncogene MYC to target genes in conjunction with WDR5^74,75^, and regulates p53 targeting gene expression^76^. Future studies on ASH2L IDR and IDR interacting proteins will provide insights into the regulation of H3K4me3 heterogeneity in cells, and potentially shed light on human pathogenesis.

Finally, histone-modifying enzyme complexes usually contain multiple IDRs in both catalytic and non-catalytic subunits. Our survey indicates that IDR content can go up to 70–90% for some histone-modifying enzymes (Supplementary Table 3). Furthermore, 60% of lysine HMTs (HKMTs) contain IDRs of 80 residues or more, whereas only 20% of other annotated proteins have IDRs of similar length^77^. It suggests that IDRs in the histone-modifying enzymes may have especially important regulatory roles, which may constitute a layer of complexity in epigenetic regulations. Inclusion of the IDRs in enzymes or enzyme complexes may be necessary to discover their regulation to the fullest extent.

## Methods

### Mouse and human ES cell lines

E14tg2a (E14) (ATCC, #30-2002) cell line was used for all cellular experiments. To generate the E14 cell line stably expressing HA-ASH2L, the plasmid expressing ASH2L from the pPiggybac-HA vector as well as plasmids carrying PBase transposase and rTTA element were co-transfected into E14 cells by electroporation. Geneticin was added one day after transfection and selection was carried out for 10 days. Single colonies were picked and screened for stable expression of HA-ASH2L in the presence of Doxycycline.

### General protein expression and purification

All MLL1 complex subunits and their mutants were expressed using the pET-28a expression vector with N-terminal 6-histidine and SUMO tag^29^. To make ASH2L mutants for methyl assignments, codon-optimized ASH2L^202–534^ DNA (Integrated DNA Technologies) was used as a template for mutagenesis. Each Ile was changed to Leu, and each Leu or Val was changed to Ile. NEBaseChanger web tool (New England Biolabs) was used to design primers for single residue substitution. Mutant plasmids were constructed using Q5 Site-Directed Mutagenesis Kit (NEB, Cat#E0554S). All proteins were expressed in BL21(DE3) *E. coli* strain in LB media. Cells were grown initially at 37 °C till OD_600_ reached 0.6–0.8 and shifted to 20 °C after IPTG was added at a final concentration of 0.2–0.4 mM. Cells were lysed by sonication and lysates were collected after centrifugation at 32,000 × *g* for 30 min at 4 °C. The supernatant was filtered through 0.45 μm syringe filter and purified through a Ni-NTA metal-affinity column (Qiagen and Goldbio). After extensive washing with 20 mM Tris (pH 8.0), 300–500 mM NaCl, 2 mM β-mercaptoethanol, and 10 mM imidazole (washing buffer), protein was eluted stepwise at 30, 60, 90, 120, 150, 210, and 300 mM imidazole. SUMO protease was added to the pooled fractions during dialysis at 4 °C overnight. Ni-NTA purification was repeated to remove 6-histidine tag and other bacterial impurities. Proteins were further purified on a HiLoad 16/60 Superdex 75 pg or 200 pg columns (GE Healthcare Life Sciences). All MLL complex subunits and their mutants are nicely expressed and well-behaved in solution with no noticeable differences in protein stability.

### GST-fusion MLL and SET1 proteins

GST-tagged MLL (MLL1^3745^, MLL2^2490^, MLL3^4689^, MLL4^5319^) and SET1 (SET1A^1474^ and SET1B^1684^) proteins were expressed using a pGEX-parallel 1 expression vector with N-terminal GST tag and TEV cleavage sequence^31^. Plasmids were transformed and expressed in BL21(DE3) *E. coli* in LB media. Cells were grown until OD_600_ reached 0.6–0.8 when the temperature was reduced to 20 °C and, after temperature equilibration, protein expression was induced using 0.4 mM IPTG and grown for 16 h. Cells were harvested and lysed using sonication and the supernatant was collected by centrifugation at 32,000 × *g*, filtered through a 0.45 µm syringe, and loaded onto a pre-equilibrated Glutathione Sepharose 4B column (GE Healthcare Life Sciences). After several washes with 20 mM Tris HCl (pH 7.5), 300 mM NaCl, 2 mM DTT, 10% v/v glycerol (GST wash buffer), the protein was eluted off of the column using GST wash buffer with 10 mM reduced glutathione. Proteins were further purified over a HiLoad 16/60 Superdex 200 pg column (GE Healthcare Life Sciences). The purified SET domains remain soluble and stable for the in vitro assays.

### In vitro HMT assay

Mixture of stoichiometric amounts of MLL1 core proteins was used for the in vitro HMT assay. Recombinant mono-nucleosome was prepared by salt dialysis of equal molar histone octamer^78^ and 146 bp 601 DNA. The reaction was carried out in 20 μL of the HMT buffer of 20 mM Tris (pH 8.0), 50 mM NaCl, 5 mM Mg^2+^, 1 mM DTT and 10% v/v glycerol^79^. The reaction was initiated by adding 1 μL of 100 μM S-adenosyl-L-methionine and incubated at room temperature for 1 h for the NCP substrates or 4 h for the recombinant H3 substrate. The 2× SDS-PAGE sample buffer was added to quench the reaction.

### Western blotting

The histones were separated on a 10–15% polyacrylamide gel and transferred onto polyvinylidene difluoride membrane (Millipore). The membrane was blocked in blocking solution, consisting of 5% milk in 0.1% 1× Tween 20/TBS (TBST), followed by incubation at 4 ^o^C overnight with the primary antibody in blocking solution. Membranes were washed three times in TBST and incubated with the HRP-conjugated anti-mouse/rabbit secondary antibodies at room temperature for 1 h. The membrane was developed using Pierce^TM^ ECL Western Blotting Substrate (Thermo Fisher Scientific, #32106), and images were captured by ChemiDoc^TM^ Touch Imaging System (Bio-rad).

### Antibodies

The primary and secondary antibodies included: Rabbit anti-H3K4me1 (Abcam, cat # ab8895, 1:20,000), Rabbit anti-H3K4me2 (Millipore, cat # 07-030, 1:20,000), Rabbit-anti H3K4me3 (Millipore, cat # 07-473, 1:10,000), Rabbit anti-Histone H3 (Abcam, cat #ab1791, 1:20,000) and anti-Rabbit IgG Horseradish Peroxidase-linked whole antibody (GE Healthcare, cat #NA934, 1:10,000). Anti-HA (clone C29F4) rabbit monoclonal antibody (Cell signaling technology, cat #3724, 1:1000).

### Preparation of ILV ^13^CH_3_-labeled ASH2L NMR samples

The U-[^2^H] Ileδ1-[^13^CH_3_] Leu, Val-[^13^CH_3_, ^13^CH_3_] ASH2L samples were produced using a previously developed protocol^80^ with modifications. Freshly transformed single colony was inoculated into H_2_O minimal media containing 6.5 g/L Na_2_HPO_4_, 3 g/L KH_2_PO_4_, 0.5 g/L NaCl, 120 mg/L MgSO_4_, 11 mg/L CaCl_2_, 10 mg/L biotin, 10 mg/L thiamine, 30 mg/L kanamycin, 2 g/L D-glucose and 1 g/L NH_4_Cl. Cells were cultured at 37 °C until OD_600_ reaches 0.25 and harvested to remove H_2_O media. Then cells were resuspended in D_2_O (99.9%, CIL, DLM-4-1000) minimal media containing the same salts in H_2_O media in which plain glucose was replaced by D-[^2^H]-glucose (CIL, DLM-2062). Cells were cultured at 37 °C until OD_600_ reaches 0.7–0.8. The temperature was lowered to 20 °C and 70 mg/L [^13^CH_3_, 3,3-^2^H] α-ketobutyrate (Cambridge Isotope Laboratory, CDLM-7318) and 120 mg/L [3-^13^CH_3_, 3,4,4,4-^2^H] α-ketoisovalerate (CIL, CDLM-73170) were added to the culture. After 1 h, IPTG dissolved in D_2_O was added to the final concentration of 0.4 mM. Cells were cultured for another 24 h before harvesting. The labeled ASH2L proteins were purified through Ni-NTA column as described above. To prevent potential aggregation of ASH2L at high concentration^52,82^, we added 1.5 molar excess of unlabeled RbBP5 (330–363) to all ASH2L samples. Acidic residue-rich RbBP5 (330–363) alleviates potential aggregation by masking the congregated basic residues in the ASH2L SPRY domain^52^. For simplicity, we use ASH2L to refer to ASH2L/RbBP5 (330–363) since only ASH2L was labeled and examined. To examine the effect of DPY30, additional 1.2 molar excess of unlabeled dimeric DPY30 was added. All the NMR samples were concentrated and buffer exchanged into 25 mM sodium phosphate, pH 6.5, 10 mM NaCl, 0.25 mM d_10_-dithiothreitol (CIL, DLM-2622), and 1 mM NaN_3_ in 99.99% D_2_O (Aldrich Cat#151882).

### NMR spectroscopy

NMR experiments were carried out on 800 MHz Bruker Ascend spectrometer equipped with pulsed-filed gradient 5 mm inverse triple resonance TXI probe and SampleCASE with 24 sample slots. IconNMR software was used for the automated collection of mutant samples for assignment. All HMQC experiments were acquired at 25 °C. Complex points of 2048 and 256 (^1^H, ^13^C) were used for most of the experiments except for Ile mutants for assignment, for which 128 complex points in ^13^C dimension were used. The ^1^H and ^13^C carrier frequencies were placed at 4.7 and 17 ppm, respectively. Spectral width was set to 12 and 20 ppm for ^1^H and ^13^C dimensions, respectively. A recycle delay of 0.5 s was used with 32–256 scans depending on protein concentration. Residual water was suppressed by the WATERGATE method. ^13^C WALTZ-16 decoupling was employed during acquisition in the direct dimension. All spectra were processed using the NMRPipe program^81^. Gaussian broaden window and sine bell window functions were applied in ^1^H and ^13^C dimensions. NMRFAM-Sparky was used to visualize NMR spectra^78^.

### Small-angle X-ray scattering

All SAXS data were collected at the 18-ID BioCAT Beamline (Biophysics Collaborative Access Team, Advanced Photon Source, Argonne National Laboratory) using the inline SEC-SAXS configuration, in which a flow cell was connected to a ÄKTApure FPLC system (GE Healthcare). To prevent potential aggregation, a stoichiometric amount of RbBP5 peptide (330–363) was added to the ASH2L samples as described above. About 200–500 μL of 1–2 mg/mL proteins were injected to a Superdex 200 column (10 × 300 mm, GE Healthcare) pre-equilibrated with 20 mM Tris (pH 7.5), 150 mM NaCl and 1 mM DTT. The flow rate was set to 0.7 mL/min during the data collection. The scattering data were collected every 2 s with 1 s exposure during the SEC elution between 5–24 ml. After data reduction, the strongest scattering data around the protein elution peak were selected for sample scattering. Several data points with minimal scattering near the elution peak were chosen for buffer-only scattering. PRIMUS^83^ was used for data processing, including averaging scattering data, background subtraction, and calculation of the radius of gyration, *R*_g_. and the Porod Volume. The molecular weight was estimated by dividing Porod Volume by 1.6. The pair distribution function was calculated by GNOM^84^ in the GUI version of PRIMUS. For EOM analysis, a pool of 10,000 structures of ASH2L with N-terminal PHD-WH and C-terminal SPRY domains connected by the Linker and Loop IDRs was generated by RANCH^56^. The sequence of RbBP5 (330–363) was not included in the EOM analysis given its small size, well-characterized interaction with the rigid SPRY domain^52^, as well as limitation of EOM for multiple polypeptide chains^56^. GAJOE was used to select an ensemble that best fit the experimental data using a generic algorithm^56^.

### Molecular modeling of ASH2L IDRs

Human ASH2L protein consists of two domains, PHD-WH domain, and SPRY domain, that have homologous PDB structures, 3S32 (A-chains), 3TOJ, respectively. The crystal structure of yeast Bre2 determined in the COMPASS complex (PDB: 6CHG) contains the Linker and Loop IDRs. The three-dimensional (3D) model for the full-length human ASH2L protein (including PHD-WH domain, Linker-IDR and Loop-IDR regions and SPRY domain) was built by C-I-TASSER^85^ using homologous PDB structures above. C-I-TASSER is a recently proposed protein structure prediction pipeline based on the classic I-TASSER protocol^86^ with newly developed residue-residue contact predictors^87,88^. LOMETS^89^ threading is performed to align the query sequence to template structures from PDB database to extract continuous fragments. These fragments are used as initial models to assemble into full-length structure by a replica-exchange Monte Carlo (REMC) simulation guided by a composite force field consisting of deep learning-predicted contacts, template-derived distance restraints, and knowledge-based energy terms calculated by statistics of PDB database. The REMC simulation produces a variety of “decoy” conformations, which are then clustered by pairwise structure similarity^90^. The centroid of the largest cluster is refined at the atomic level by FG-MD^91^ to obtain the final C-I-TASSER 3D model. The first model generated by C-I-TASSER was selected as the ASH2L model for the following analysis. The estimated TM-score of the entire model was 0.67 ± 0.13, indicating that it was a high-confidence model^92^. We removed the PHD-WH domain from the model during the cryo-EM fitting and refinement steps, since there is no density map collected for the PHD-WH domain.

### Cryo-EM sample preparation and data collection

The GraFix method^93^ was applied to the MLL1^RWSA^- NCP complex to prepare for the cryo-EM grid. In brief, 30 μM of MLL1^RWSA^ was incubated with 10 μM NCP and 0.5 mM *S*-adenosyl-L-homocysteine for 30 min at 4 °C in the GraFix buffer (50 mM HEPES, pH 7.5, 50 mM NaCl, 1 mM MgCl_2_, and 1 mM TCEP). The sample was centrifuged at 100,000 × *g* at 4 °C for 3 h after applying onto a centrifuge tube, which contained a gradient solution of 0–60% glycerol and 0–0.2% glutaraldehyde. After centrifugation, the crosslinked sample was quenched with 1 M Tris-HCl, pH 7.5. To remove glycerol from the GraFix buffer, we performed further buffer exchange using a centrifugal concentrator (Sartorius Vivaspin 500).

The sample at ~1 mg/ml was applied onto a glow discharged Quantifoil R1.2/1.3 grid (Electron Microscopy Sciences) at 4 °C with 100% humidity. The loaded grid was plunged-frozen in liquid ethane after 4 s blotting and 30 s waiting using a Mark IV Vitrobot (Thermo Fisher Scientific). The cryo-EM data were collected using Titan Krios (Thermo Fisher Scientific) operating at 300 keV with the K2 Summit direct electron detector. The movie data was recorded in a counting mode at a ×29,000 magnification and the pixel size of 1.01 Å/pixel, with a defocus range between −1.5 to −2.5 μm. A dose rate of 1.28 electrons/Å^2^/frame with a total 50 frames per 8 s was applied for data collection, resulting in a total dose of 64 electrons per Å^2^. A total of 6,242 movies were collected.

### Cryo-EM data processing and model refinement

Micrograph movies were aligned with whole-frame and local drift correction using MotionCorr2^94^, and CTF was estimated with CTFFIND4.1^95^. Micrographs with higher than 4.5 Å of the estimated resolution were further selected, which resulted in 6137 micrographs. A total of 1,287,771 particles were picked using Warp^96^. The particles were extracted in RELION^58^ and imported into cryoSPARC^57^ for 2D classification. After excluding bad particles, a total of 1,194,542 particles were subjected to the first round of ab initio 3D classification into five classes (Supplementary Fig. 4). Two of five classes were subjected to the second round of ab initio 3D classification into five subclasses, and the subsequent heterogeneous refinement was performed. Four of the five subclasses displayed a well-defined map of the MLL and nucleosome complex after the heterogeneous refinement. They were exported for 3D classification. The focused 3D classification was performed at the MLL1^RWSA^ region without alignment (35 cycles, *T* = 4, binary mask: 10 pixels/soft mask: 10 pixels). The Class03 was excluded because it displayed a structurally heterogeneous and unresolvable EM density even after the focused 3D classification. The best behaving class selected from Class01 (13,086 particles), Class02 (27,730 particles), and Class05 (23,236 particles) was subjected to the 3D auto refinement and further post-processed to a resolution of 6.9, 4.6, and 6.0 Å, respectively. Each final cryo-EM map was locally filtered to avoid over-estimation. The resolution of all structures was estimated by RELION with Fourier shell correlation (FSC) at the criteria of 0.143.

For the model building, the rigid-body fitting was performed for each class using Chimera^97^. The cryo-EM structure of MLL1^RWSAD^-NCP (PDB ID: 6PWV [10.2210/pdb6pwv/pdb])^98^ was used for the rigid-body fitting for each individual class. For the model refinement, each class was subjected to the real-space refinement using PHENIX^99^, and model validations were performed by MolProbity^100^. Statistics for data collection, refinement, and validation were summarized in Supplementary Table 2.

### ESC culture and transfection

E14tg2a (E14) (ACTT, #CRL-1821TM) were grown in the KnockOut™ DMEM medium containing 15% FBS, 2 mM glutamine, 1X non-essential amino acids, 0.1 mM 2-mercaptoethanol and 10^3^ U ml^−1^ LIF (Millipore, #ESG1107), unless otherwise indicated. E14 cells were routinely tested for negative mycoplasma contamination using the LookOut® Mycoplasma PCR Detection Kit (SIGMA ALDRICH, #MP0035) according to the manufacturer’s instructions. For expressing dCas9 fusion proteins, E14 ESCs were transfected with pcDNA3-dCas9-HA and pcDNA3-dCas9-DPY30-HA plasmids using Fugene 6 (Promega, Cat# E2691) for 2 days and then selected with G418 (400 µg/ml, Gibco, Cat# 10131-035) for 5 days. After selection, the cells were split and transfected with a pool of three pspgRNA-gRNAs for selected genomic loci. The pspgRNA-gRNAs were co-transfected with a pBase vector (1:10) that confers puromycin resistance. After 2 days of puromycin selection (1.5 µg/ml, Gibco, Cat# A11138-03), the cells were subject to ChIP using anti-HA antibody (Cell Signaling Technology, cat# 3724) and anti-H3K4me3 antibody (Millipore, Cat# 07-473), respectively. ChIP-qPCRs were performed to detect the enrichment of H3K4me3 and HA in each location.

### CUT&RUN

CUT&RUN was performed according to the protocol described previously^101^. HA-ASH2L E14 and the parental E14 (E14tg2a, ACTT, #CRL-1821TM) cell lines were cultured in presence of 1 μg/mL Doxycycline for 2 days. Biological duplicates were performed for HA-ASH2L and H3K4me3, respectively. For each experiment, 1 × 10^6^ cells were harvested, washed with wash buffer (20 mM HEPES pH7.5, 150 mM NaCl, 0.5 mM spermidine, 1× protease inhibitor cocktail), and incubated with Concanavalin A-coated beads (Bangs Laboratories, Inc. #BP531) for 15 min with rotation. Bead-bound cells were resuspended in solution (digitonin/wash buffer) and incubated with anti-HA (Cell Signaling, #3724) or anti-H3K4me3 (Millipore, #07-473) antibodies overnight at 4 °C. The beads were washed with digitonin/wash buffer three times before adding protein A-MNase (0.5 ng/μL) and incubating for 1 h at 4 °C. Following three washes, bound protein A-MNase was activated on ice for 30 min by the addition of 3 mM CaCl_2_. The reaction was quenched with equal volume of 2× stop buffer (340 mM NaCl, 20 mM EDTA, 4 mM EGTA, 0.02% Digitonin (EMD Millipore #300410), 50 μg/mL RNase A (QIAGEN #19101), 50 μg/mL glycogen (Roche #10901393001), 2 pg/mL Drosophila spike-in DNA) at 37 °C for 30 min. The proteins were removed by incubating with 0.1% SDS and 0.15 mg/mL Proteinase K (Roche 3115879001) at 65 °C for 2 h. DNA fragments were purified by phenol-chloroform and ethanol precipitation and subjected to library preparation. The sequencing was performed at the University of Michigan Advance DNA Sequencing Core.

### ChIP analysis and quantitative real-time PCR (qPCR)

E14 cells expressing dCas9 fusion proteins were transfected with or without pooled gRNAs (4~5 gRNAs for each selected region) (Supplementary Table 4) prior to the experiment. Cells were crosslinked with 1% paraformaldehyde at room temperature for 10 min and quenched by 250 mM glycine. After two washes with cold 1×PBS, cells were lysed, and the chromatin was sonicated for three times for 20 min each using Diagenode Bioruptor 300 for 3 rounds of 20 cycles with 30” on/off per cycle. The supernatant of the sonicated lysate was diluted with 5 volumes of ChIP dilution buffer (16.7 mM Tris-HCl pH 7.5, 12 mM EDTA, 1.1% Triton X-100, 167 mM NaCl, 0.01% SDS) and incubated with anti-H3K4me3 or anti-HA antibodies at 4 °C overnight. The immune complexes were purified on 30 µl of protein G magnetic beads (Invitrogen, Cat# 10003D) for 2 h at 4 °C, followed by three times of washes with low stringency buffer (50 mM HEPES pH 7.9, 5 mM EDTA pH 8.0, 1% NP-40, 0.2% DOC, 1×PBS) and high stringency buffer (50 mM HEPES pH 7.9, 5 mM EDTA pH 8.0, 1% NP-40, 0.7% DOC, 500 mM LiCl) as well as two times washes with Last Wash Buffer (5× TE pH 8.0, 0.3% NP-40). The beads were eluted twice with elution buffer (100 mM NaHCO3, 1% SDS) and reverse-crosslinked at 65 °C overnight. The samples were incubated with RNAse A at 37 °C for 30 min, followed by incubation with Proteinase K (20 mg/ml) at 45 °C for 1 h. DNA was recovered by phenol-chloroform extraction and ethanol precipitation. Real-time PCR was carried out using Radiant Green 2× QPCR mix (Alkali Scientific, Cat# QS1050) on Bio-Rad Real-time PCR machine. Primer information for real-time PCR is included in Supplementary Table 4.

### ChIP-seq data mapping and normalization

ChIP-seq dataset for DPY30 and MLL1 were downloaded from GEO GSE26136 and GEO GSE107406, respectively. Paired-end sequencing reads were trimmed with trim_galore to remove adaptor sequences. We kept reads that were 20 bp or longer after trimming and paired between the mates. All ChIP-seq data were mapped to the mouse mm10 genome by using Bowtie2 (v2-2.2.4)^102^ with parameters “-q --phred33 --very-sensitive -p 10”. Duplicated reads were removed using SAMtools (v1.5)^103^. The bigwig files for IP/input ratio were generated from BAM files by using deepTools3 (v3.2.1)^104^ with command “bamCompare -b1 ChIP-bam -b2 Input-bam --ignoreDuplicates --minMappingQuality 30 --normalizeUsing RPKM --binSize 1 --operation ratio --scaleFactorsMethod None -p 20”. BAM files for mapping results were merged using SAMtools and converted to BED format using BEDTools^105^. Peaks were called from bed files using MACS (v 1.4.2)^106^ with parameters “-w -S -p 0.00001 -g mm”. The input signal was used as the control for peak calling. Heatmap of ChIP-seq signals were visualized using deepTools3.

### CUT&RUN peak calling and visualization

HA or H3K4me3 CUT&RUN from two independent biological replicates were initially analyzed in parallel. Paired-end sequencing reads were processed as described above. The resulting alignments, recorded in BAM file, were sorted, indexed, and marked for duplicates with SAMtools^103^. The analysis showed a good correlation and signal-noise ratio from replicates. The BAM files for mapping results from the replicates were used for further analysis. The overlapping peaks were merged as the union of all using SAMtools and converted to BED format using BEDTools^105^. Fragments with size <120 bp were retained^107^ by using subcommand “alignmentSieve” in deepTools3^104^. Peaks were called from bed files using MACS (v 1.4.2)^106^ with parameters “-w -S -p 0.00001 -g mm”. The bigwig files for visualization were generated from MACS. Heatmap of CUT&RUN signals were visualized using subcommand “computeMatrix” and “plotHeatmap” in deepTools3.

### Statistical analysis and reproducibility

Statistical analysis was performed by two-tailed Student’s *t*-test using GraphPad Prism 7.0 software. Data were presented as the standard error of the mean (SEM). *p* value of <0.05 was considered statistically significant; *p < 0.05, **p < 0.01, ***p < 0.001. For all in vitro HMT experiments shown Figs. 1–3 as well as Supplementary Figs. 1, 2, and 11, a minimum of three independent experiments were performed and consistent results were obtained.

### Reporting summary

Further information on research design is available in the Nature Research Reporting Summary linked to this article.

## Supplementary information

Supplementary Information

Reporting Summary

## Data Availability

The data that support this study are available from the corresponding author upon reasonable request. ChIP-seq datasets for HA-ASH2L and H3K4me3 generated in this study are accessible at GEO with accession code GSE146933. DPY30 and MLL1 datasets were downloaded from GEO GSE26136 and GEO GSE107406, respectively. Cryo-EM structures for the 4-MLL1-NCP reported in this study are available with accession numbers: Class01 – PDB 6W5I [10.2210/pdb6w5i/pdb] and EMDB EMD-21542; Class02 – PDB 6W5M: [10.2210/pdb6w5m/pdb] and EMDB: EMD-21543; Class05 – PDB 6W5N: [10.2210/pdb6w5n/pdb] and EMDB: EMD-21544. Source data are provided with this paper.

## Source data

Source Data
