# Supplementary material for: Mechanism for DPY30 and ASH2L intrinsically disordered regions to modulate the MLL/SET1 activity on chromatin
Source: Nat Commun. 2021 May 19;12:2953. doi: 10.1038/s41467-021-23268-9 (PMC8134635; doi:10.1038/s41467-021-23268-9)
Supplement: Supplementary file 1 — Supplementary Information [file 41467_2021_23268_MOESM1_ESM.docx]

**SUPPLEMENTARY INFORMATION**

**Mechanism for DPY30 and ASH2L Intrinsically Disordered Regions to Modulate MLL/SET1 Activity on Chromatin**

Young-Tae Lee, Alex Ayoub, Sang-Ho Park, Liang Sha, Jing Xu, Fengbiao Mao, Wei Zheng, Yang Zhang, Uhn-Soo Cho, and Yali Dou

**SUPPLEMENTARY FIGURES**


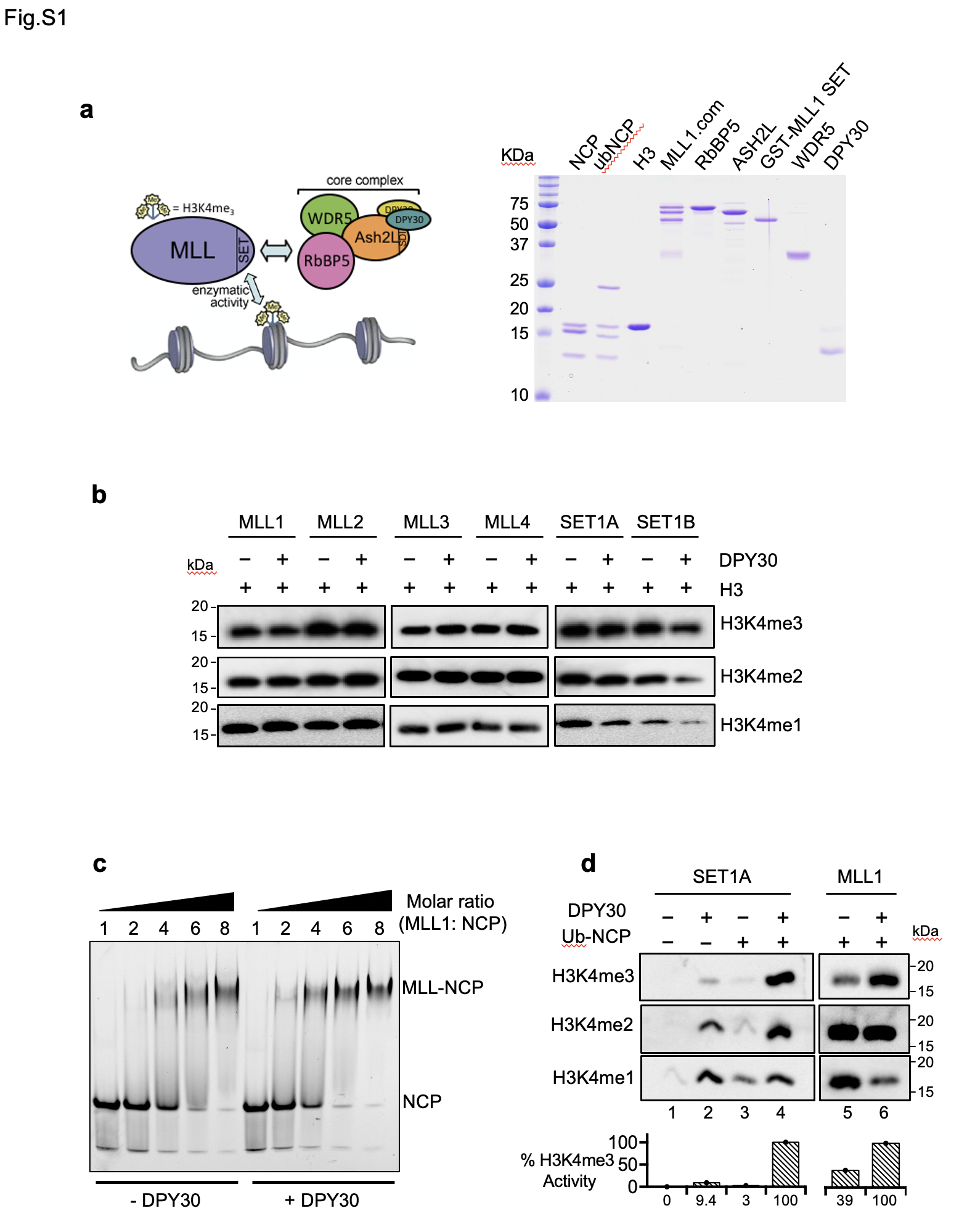


**Supplementary Figure 1 | DPY30 stimulates MLL1/SET1 activity on the NCP.** This figure is related to main Figure 1. **a**, Left, cartoon model of the subunits that comprise the MLL core complex. Full-length components are represented here and used in experiments with the exception of MLL1 protein, where only the SET is used in the HMT assay. Right, Coomassie brilliant blue staining of recombinant proteins used in this study including the NCP, the H2BK120ub-containing NCP, recombinant histone H3, the MLL1 core complex and individual components of the MLL1 core complex as indicated on top. **b**, DPY30 does not affect MLL1 activity on recombinant H3. *In vitro* HMT assay for the MLL/SET1 family histone methyltransferases using recombinant H3 as the substrate. The reactions were carried out for longer time than those on the NCP and the immunoblots were subject to a longer exposure due to weaker enzymatic activity of these enzymes on recombinant histone H3. Antibodies used for immunoblots were indicated on right. **c**, DPY30 does not affect binding of the MLL1 complex to the NCP. Electrophoretic mobility shift assay of the MLL1 core complex binding to nucleosome in the presence or absence of DPY30. Molar ratio of the MLL1 complex to NCP was indicated on top. NCP concentration was 0.4 µM. **d**, DPY30 and H2BK120ub stimulate SET1 and MLL1 activity through distinct mechanisms. *In vitro* HMT assay for the SET1A and the MLL1 core complexes on unmodified NCP (denoted as ‘-‘) or H2BK120ub-NCP (‘+’) substrates as indicated on top. Quantification of H3K4me3 was done using ImageJ ^1^ and presented as relative %activity to that of lane 4 and lane 6, respectively.

**
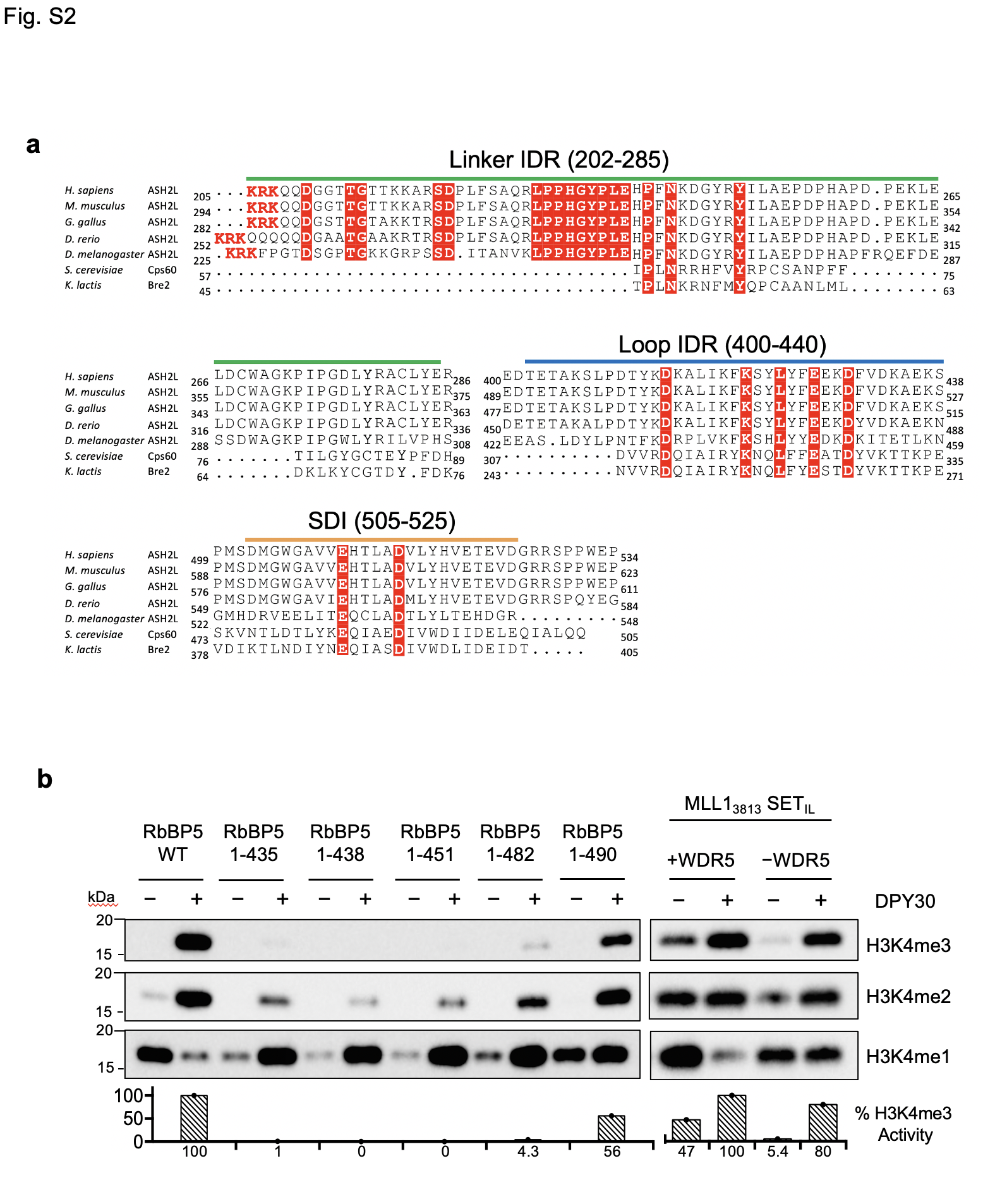
Supplementary Figure 2 | IDRs in RbBP5 and MLL1^SET^ are not essential for DPY30-dependent regulation.** This figure is related to main Figure 2 and Figure 3. **a**, Sequence alignment for eukaryotic ASH2L and ASH2L homologs. Highly conserved sequences are highlighted in red. IDR regions of interest (i.e., Linker, Loop, and SDI) are annotated. **b**, *In vitro* HMT assay for the MLL1 complex containing RbBP5 truncation fragments (left) or the MLL13813^SET N3861I/Q3867L^ (MLL^SETIL^) with or without WDR5 (right) as indicated on top. RbBP5 has an N-terminal WD40 repeat (1-381aa) and a C-terminal IDR (382-538aa). We serially deleted RbBP5 from C-terminus to gradually removed C-terminal IDRs. The assays were performed in the presence or absence of DPY30. Antibodies used in the immunoblots were indicated on right. Quantification of H3K4me3 for reaction containing DPY30 was done using ImageJ ^1^ and presented as relative %activity to that of MLL1 complex with wild type RbBP5.

**
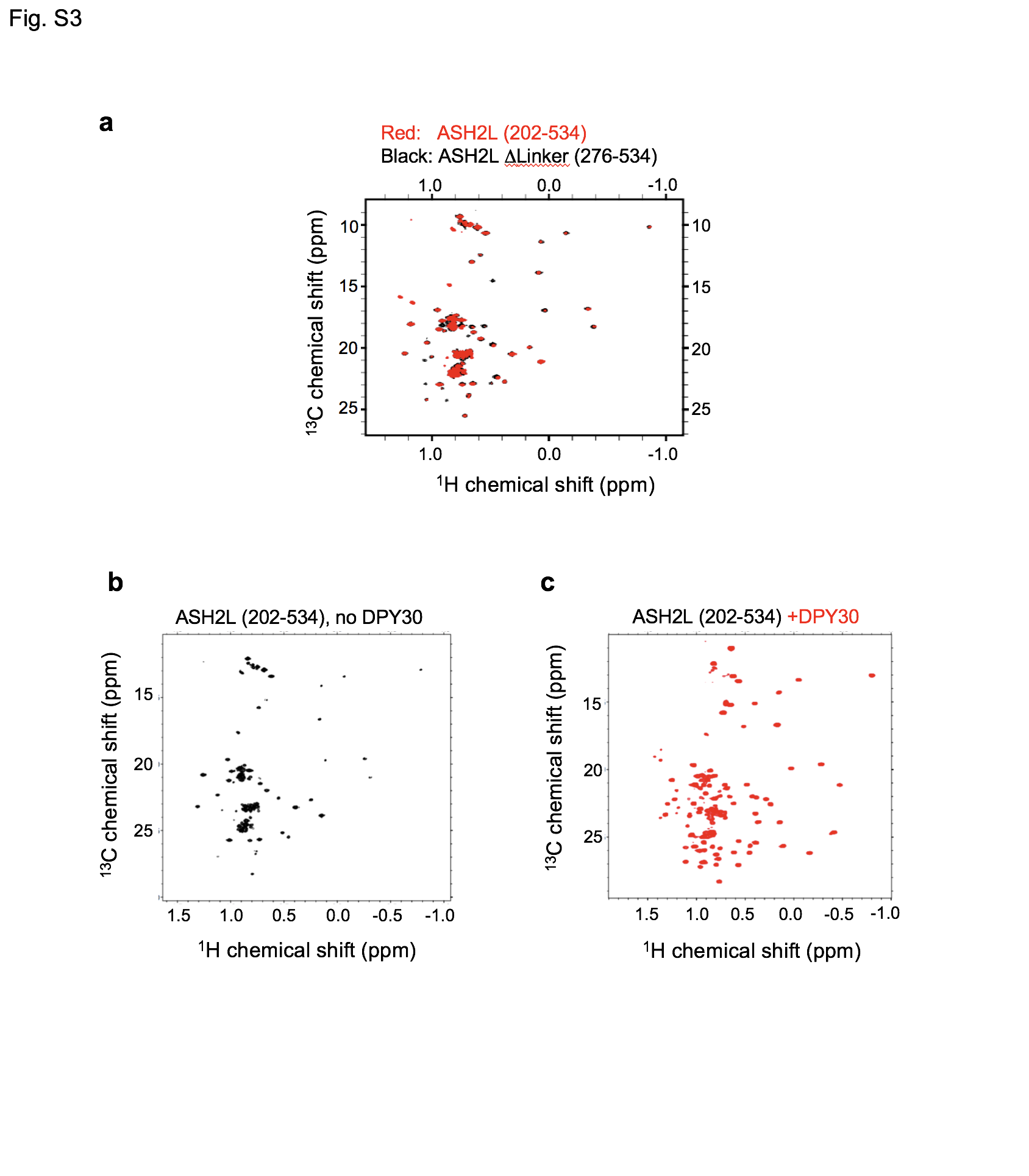
**

**Supplementary Figure 3 | NMR spectra for ASH2L.** This figure is related to main Figure 4a. **a**, Linker IDR does not have NMR spectra in the apo-state of full length ASH2L. Methyl-TROSY spectra of [^2^H, ^13^CH_3_-ILV] ASH2L^202-534^ (red) and [^2^H, ^13^CH_3_-ILV] ASH2L^ΔLinker,276-534^ (black) were superimposed. **b-c**, ASH2L spectra undergo drastic changes upon addition of DPY30. **b**, Methyl-TROSY spectrum of [^2^H, ^13^CH_3_-ILV] ASH2L^202-534^ in the absence of DPY30. **c**, Methyl-TROSY spectrum of [^2^H, ^13^CH_3_-ILV] ASH2L^202-534^ in complex with DPY30. Stoichiometric ratio between ASH2L and homodimeric DPY30 was 1:1.2.

**
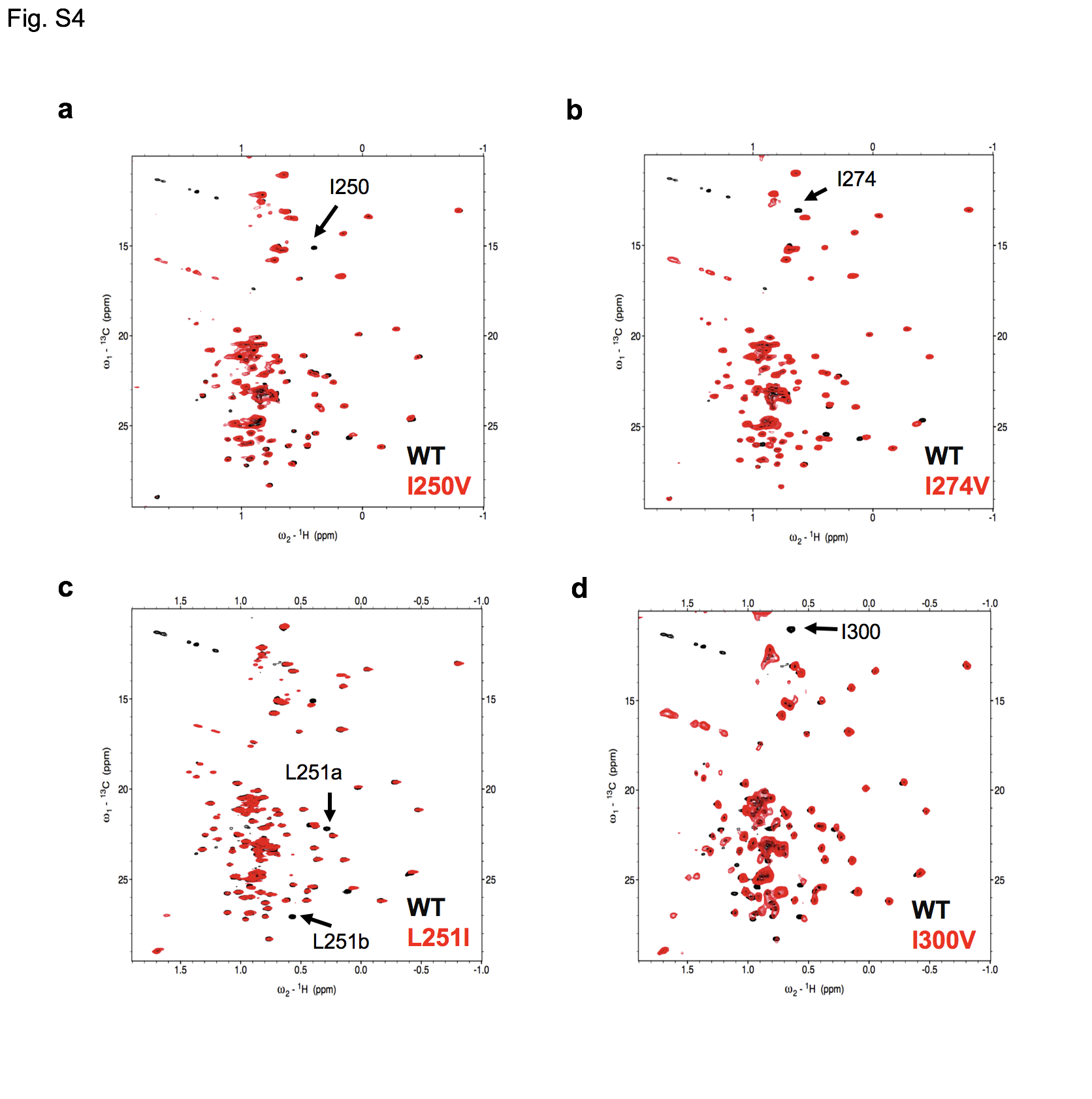
**

**Supplementary Figure 4 | Examples for the residue-specific mutagenesis assignment by methyl-TROSY approach.** This figure is related to main Figure 4a. **a**, Superimposed methyl-TROSY spectra of DPY30-bound [^2^H, ^13^CH_3_-ILV] wild-type ASH2L^202-534^ (black) and single residue mutant ASH2L^202-534, I250V^ constructs (red). Disappeared peak was assigned to the mutated residue. **b**, I274V, **c**, L251I and **d**, I300V were examined for residue-specific mutagenesis assignment by methyl-TROSY. Using this approach, 65% of peaks were assigned without ambiguity.

**
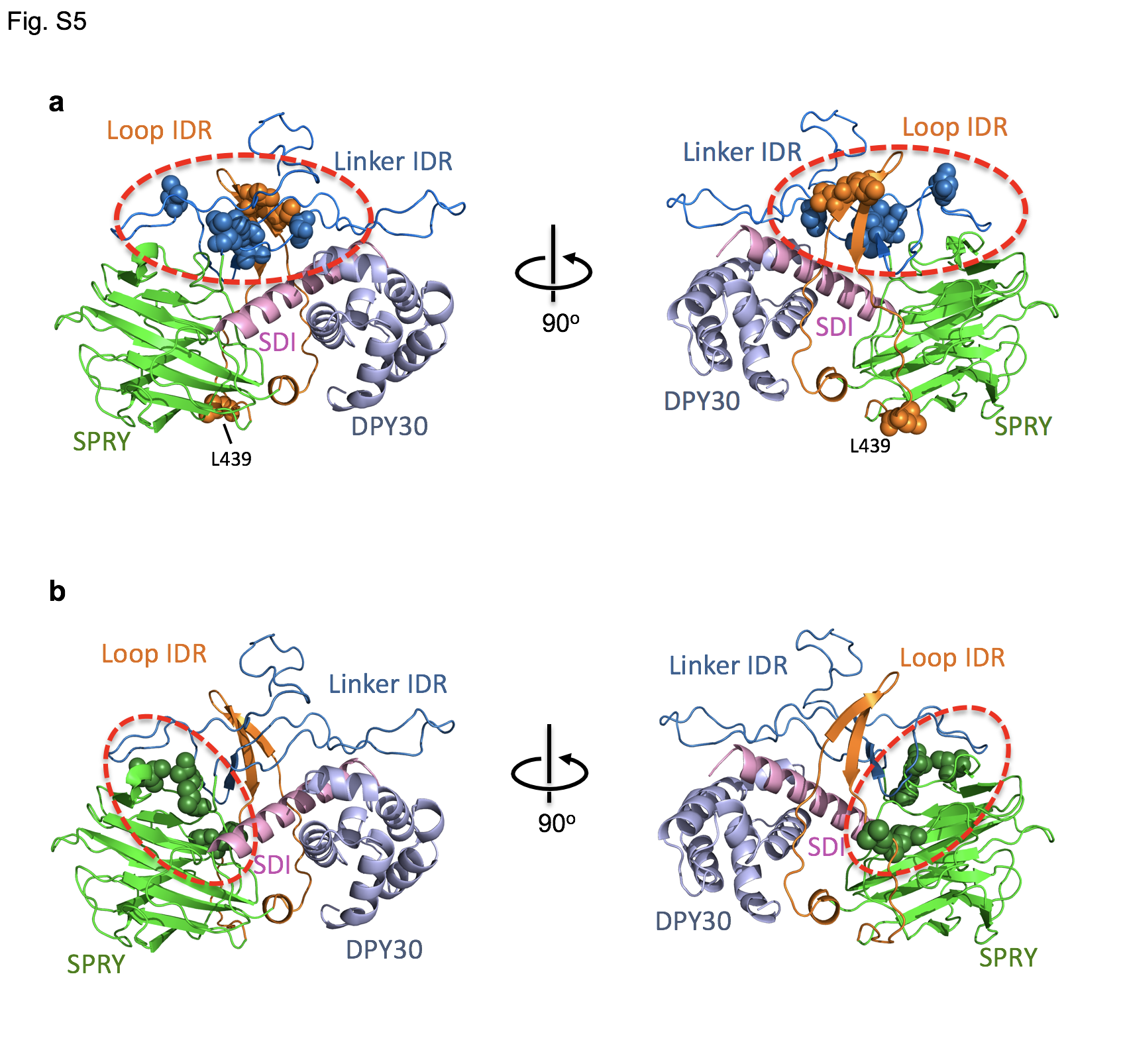
**

**Supplementary Figure 5 | Detailed molecular models that highlight ASH2L IDRs and SPRY regions that undergo DPY30-induced changes in NMR spectra.** This figure is related to main Figure 4b. Newly appeared peaks are highlight by sphere representation in the ASH2L-DPY30 structural model. SPRY, green; Linker IDRs, blue; Loop IDRs, orange; SDI, pink. Different viewpoints for **a**, IDR residues and **b**, SPRY residues are shown.

**
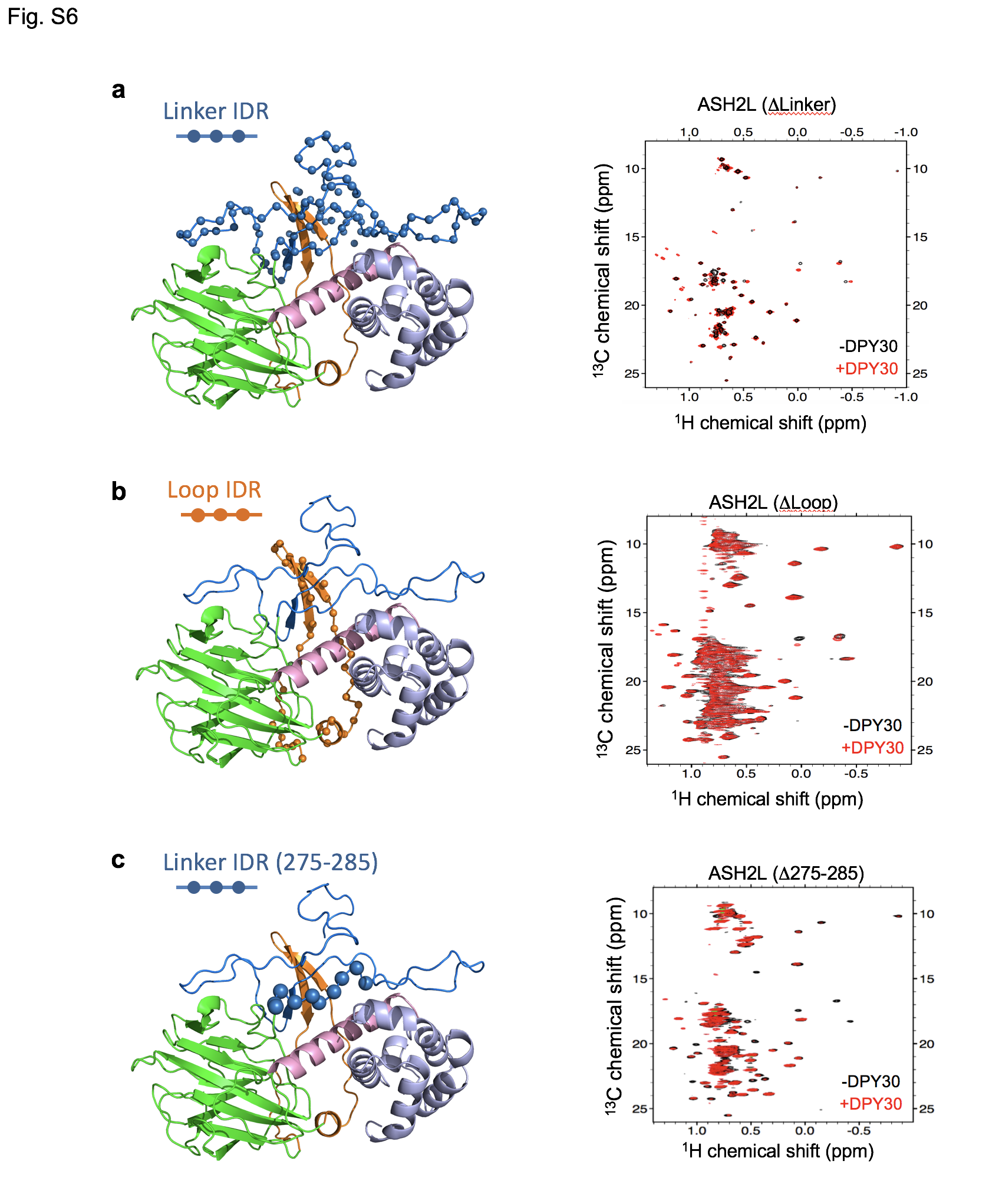
**

**Supplementary Figure 6 | Deletion of ASH2L IDR segments abolished DPY30-induced changes in NMR spectra.** This figure is related to main Figure 4. **a-c**, Left, computational models that highlight specific ASH2L IDRs in a spherical representation. **a.** Link IDR; **b.** Loop IDR; **c.** residues 275-285 in Linker IDR. Right, superimposed Methyl-TROSY NMR spectra of [^2^H, ^13^CH_3_-ILV] ASH2L^202-534^ with a designated deletion (indicated on top) in absence (black) or presence of homodimeric DPY30 (red). Compared to wild type ASH2L^202-534^, most DPY30-induced changes were abolished in the ASH2L mutants. SPRY, green; Linker IDRs, blue; Loop IDRs, orange; SDI, pink.

**
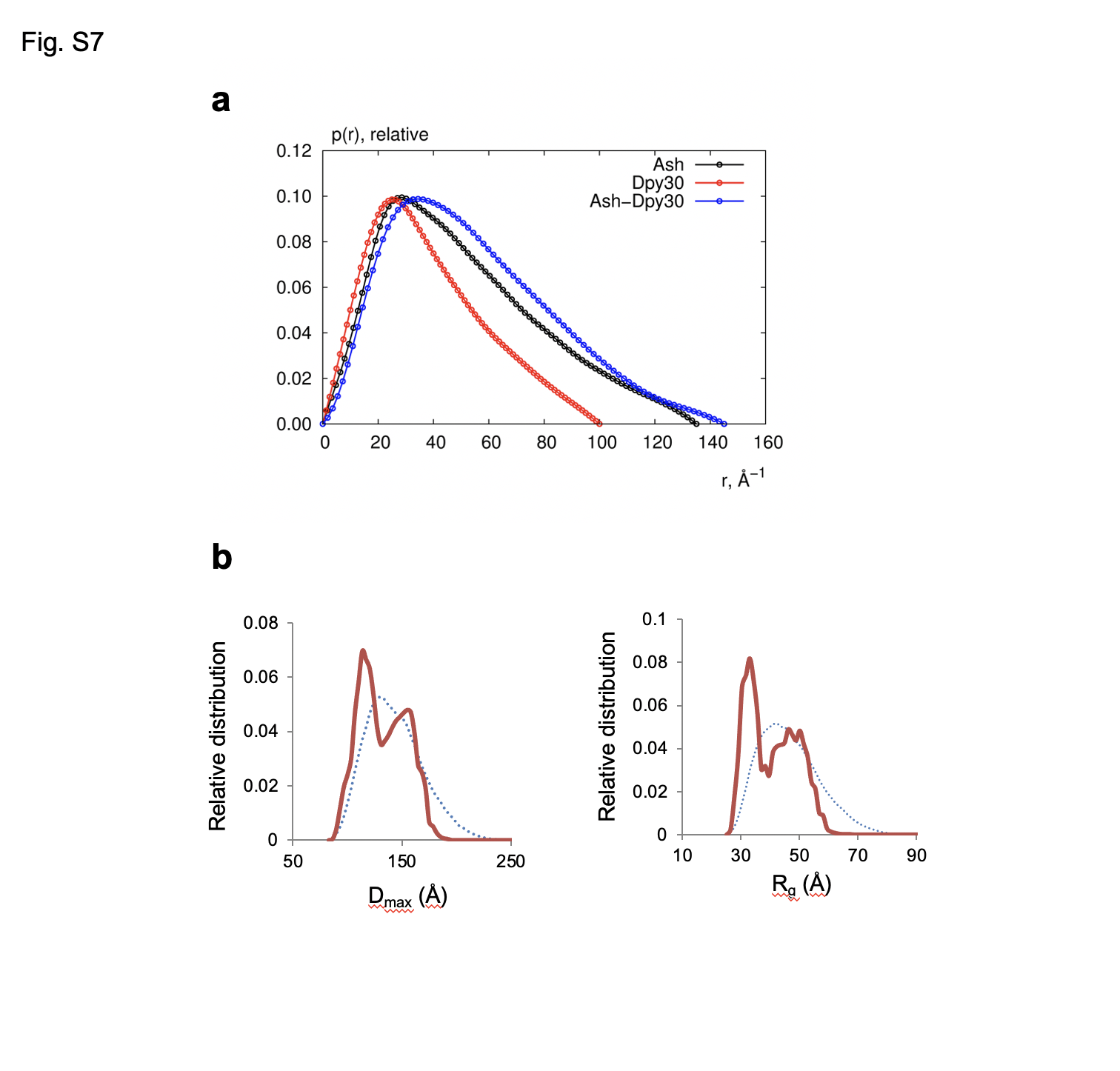
**

**Supplementary Figure 7 | SAXS studies on the free ASH2L, DPY30 and DPY30/ASH2L**. This figure is related to main Figure 4. **a,** Pair distance distribution P(r) functions of ASH2L, DPY30 and the ASH2L-DPY30 complex. **b,** Ensemble Optimized Method (EOM) analyses for free ASH2L. Distribution of a pool of 10,000 structures (blue) and optimally fit ensemble (red) are plotted against D_max_ (left) and R_g_ (right).

**
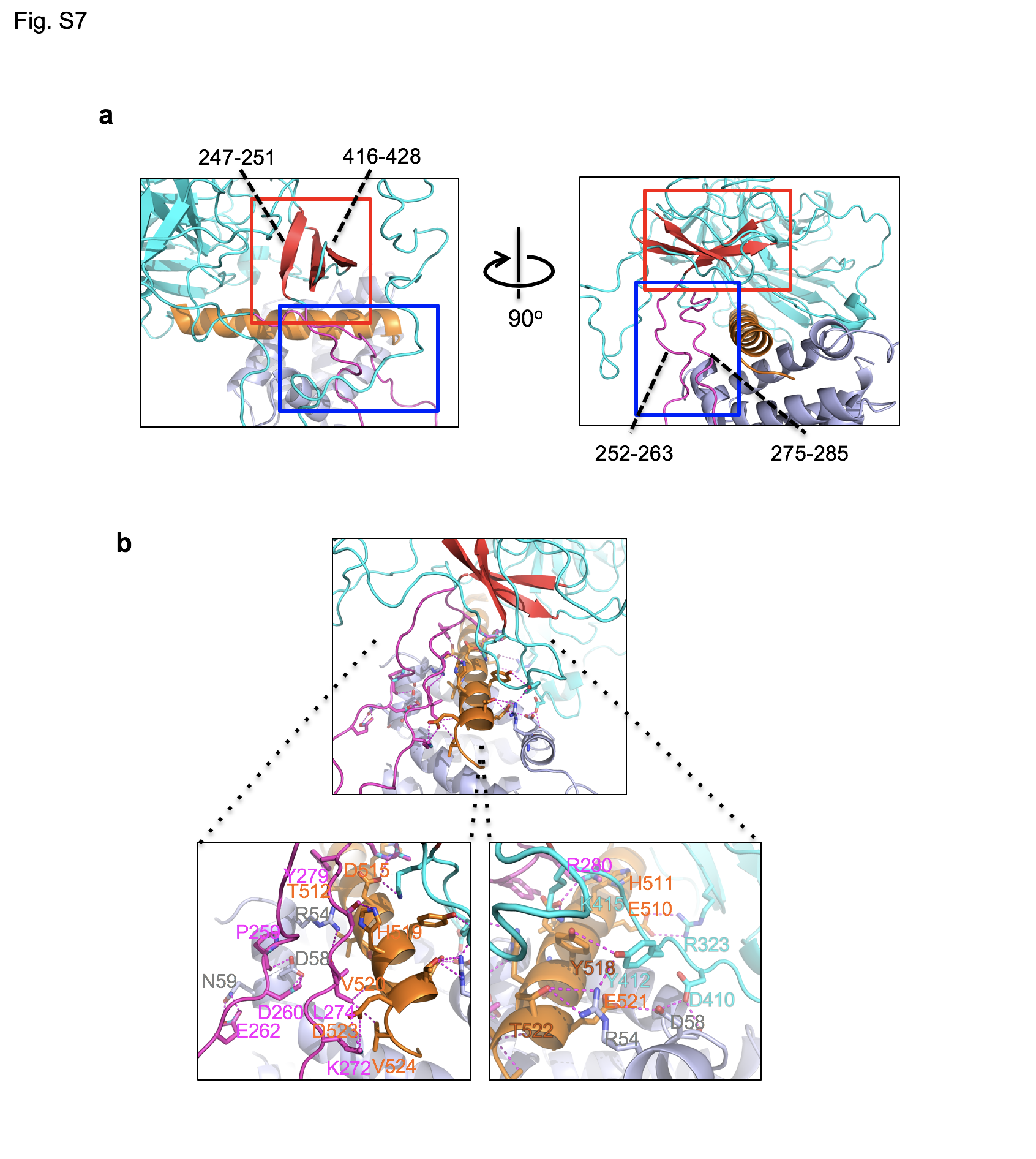
**

**Supplementary Figure 8 | The computational model for ASH2L IDR residues that are important for *in vitro* HMTs activities.** This figure is related to main Figure 1-4. **a**, The computational model shows that ASH2L Linker and Loop IDRs likely form a three-strand β-sheet (left) and a β-like structures upon DPY30 interaction. The β-sheet, β-like structure, SPRY domain, SDI of ASH2L as well as DPY30 were labeled in red, pink, cyan, orange and purple, respectively. **b**, The computational model shows likely interactions among residues in ASH2L IDRs and the α-helical ASH2L SDI. The enlarged interaction interface was shown on bottom. Characteristic secondary structures of ASH2L (β-sheet and β-like, α-helical ASH2L SDI) were shown in red, pink and orange, respectively. Residues that make potential direct contacts in the model were highlighted. Notably, some of the highlighted ASH2L residues were tested for MLL1 methyltransferase activity on the NCP in main Figure 1-3.

**
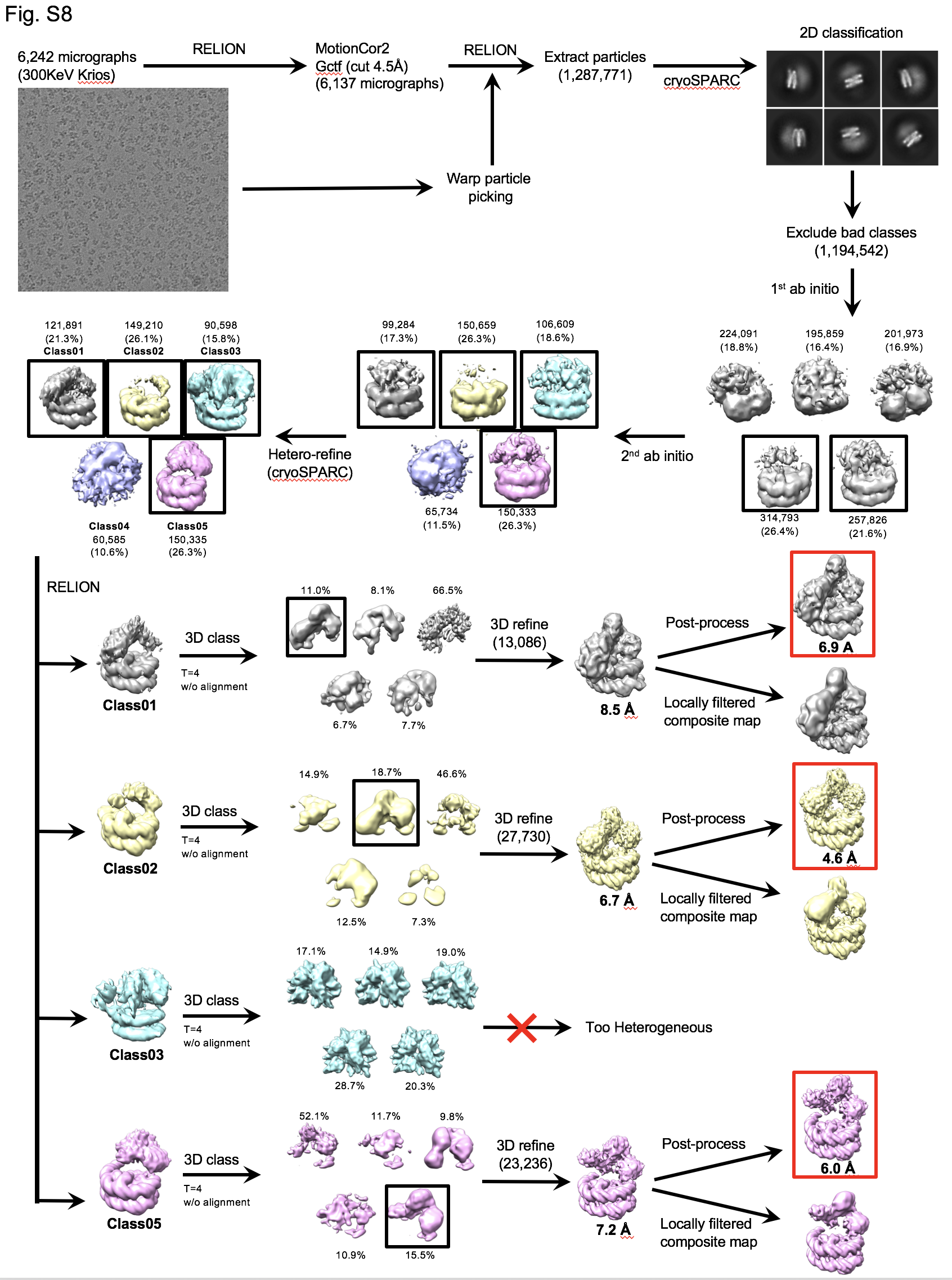
**

**Supplementary Figure 9 | Cryo-EM data processed for 4-MLL1-NCP complex.** This figure is related to main Figure 6. Representative micrograph image (Titan Krios 300 KeV) and 2D classifications of the 4-MLL1-NCP complex. The number of particles for each classification and an estimated resolution for overall and selected subcomplexes (red box) were provided. Additional data processing information can be found in the Methods.

**
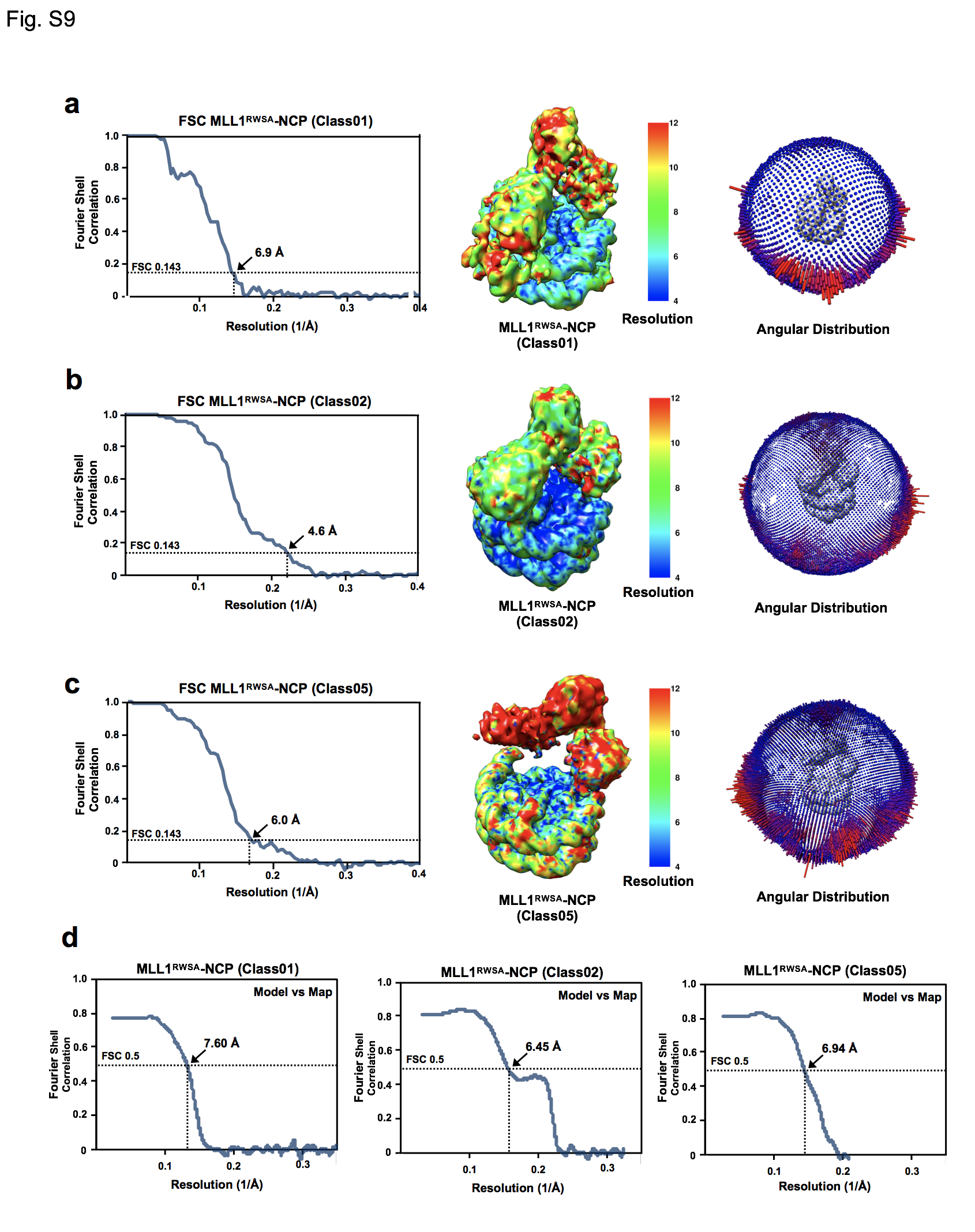
**

**Supplementary Figure 10 | Cryo-EM map validation of 4-MLL1-NCP classifications.** This figure is related to main Figure 6. Fourier Shell Correlation (FSC) curves (left), the corresponding local resolution assessment by RESMAP (middle) ^2^, and angular distribution plots (right) for the 4-MLL1-NCP **a** (Class01), **b** (Class02), and **c** (Class05) particles. The final resolution was determined using FSC = 0.143 criterion, represented by an arrow on each FSC curve. **d**, Model-map FSC curves for 4-MLL1-NCP Class01, 02 and 05 were calculated using phenix.mtriage ^3^. The resolution was found using FSC = 0.5 criterion as indicated by an arrow on each FSC curve.


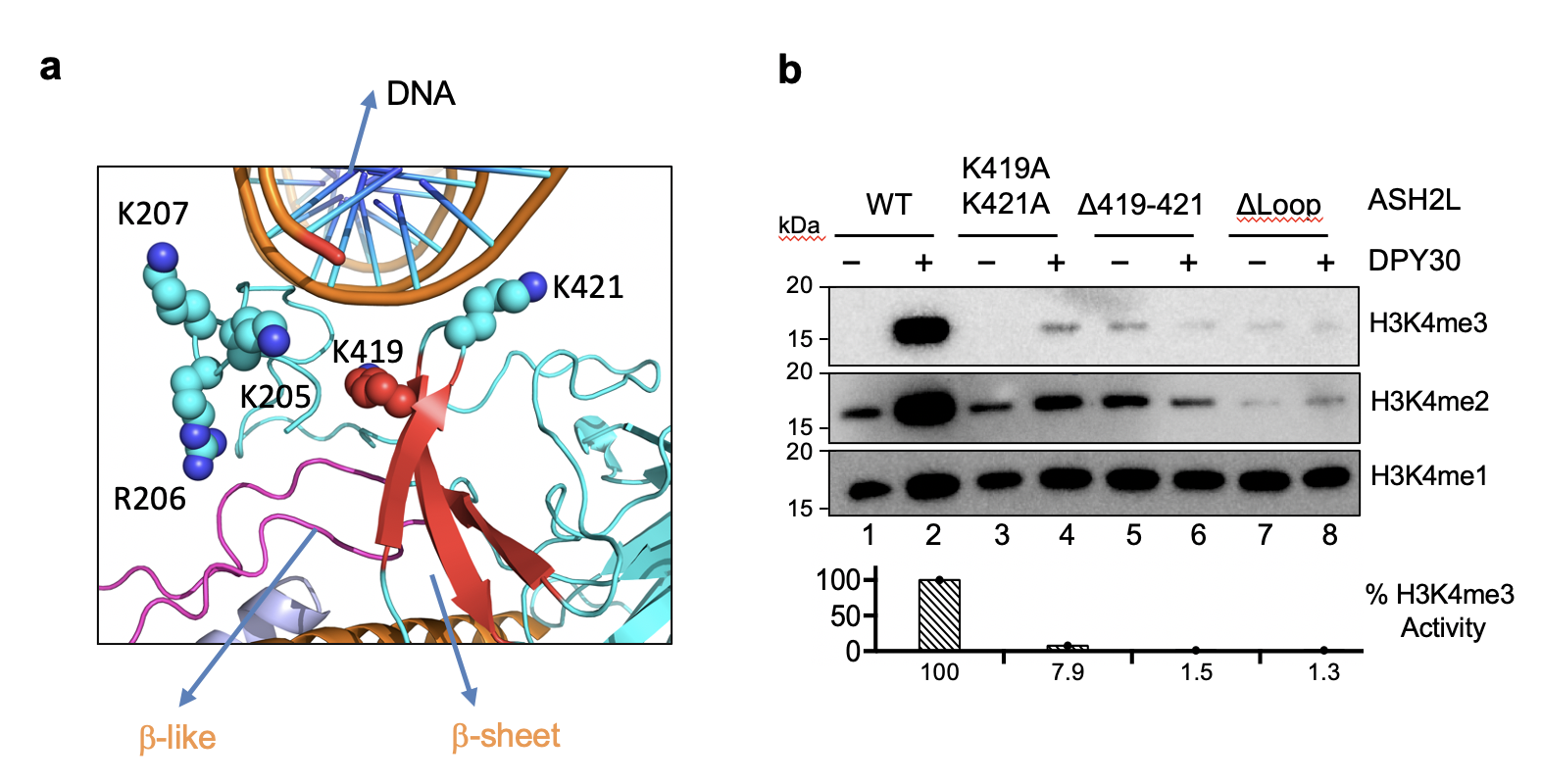


**Supplementary Figure 11 | Computational model predicts new contacts between ASH2L IDRs and the NCP.** This figure is related to main Figure 5. **a**, Molecular modeling shows that DPY30-induced conformational change in ASH2L IDRs may enable a short loop (_419_-KFK-_421_) in the Loop IDR to interact with nucleosomal DNA. Cryo-EM structure of the 5-MLL-NCP structure (PDB ID: 6PWV and EMDB: EMD-20512) ^4^ was used for the modeling. Red, DPY30-induced β-sheet structure from ASH2L Linker and Loop IDR; pink, β-like structure from ASH2L Linker IDR; orange, DNA (top). In this model, basic ASH2L residues K419 and K421 are positioned near nuclear DNA. **b**, *In vitro* HMT assay for the MLL1 core complex containing wildtype (WT) or mutant ASH2L proteins as indicated on top. The assays were performed in the presence or absence of DPY30. Antibodies for detection of the methylation products were indicated on right. Mutation or deletion of basic residues in Loop IDR, predicted by the model, drastically reduced MLL1 activity on H3K4me3. Quantification for samples containing DPY30 was performed using ImageJ ^1^ and presented as relative %activity to that of WT ASH2L.

**
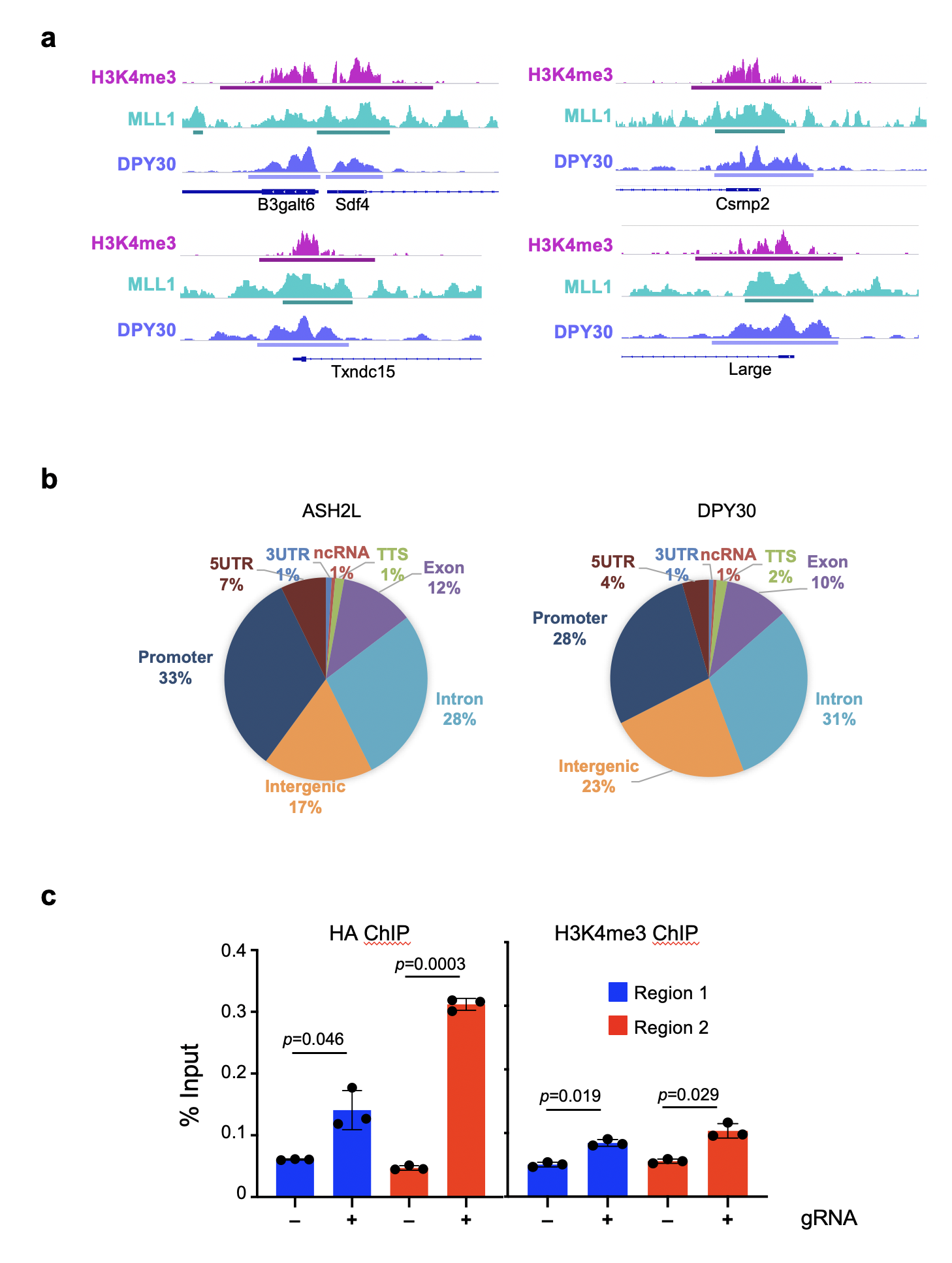
**

**Supplementary Figure 12 | *In vivo* analysis of DPY30 and ASH2L binding and H3K4me3.** This figure is related to main Figure 6. **a**, UCSC browser views of H3K4me3, MLL1 and DPY30 tracks at two randomly selected genomic loci. Peaks called by MACS2 were highlighted on bottom. **b**, Pie charts for distribution of ASH2L (left) and DPY30 (right) binding sites relative to annotated gene structures. **c**, The ChIP assay for HA (left) or H3K4me3 (right) in HA-dCas9 cells transfected with or without the pooled gRNAs. This experiment serves as the control for Figure 6b. ChIP signals were normalized against input and presented as %Input. Means and standard deviations (error bars) from at least three independent experiments were presented. Two-sided student *t* test was performed to calculate *p*-value.

**
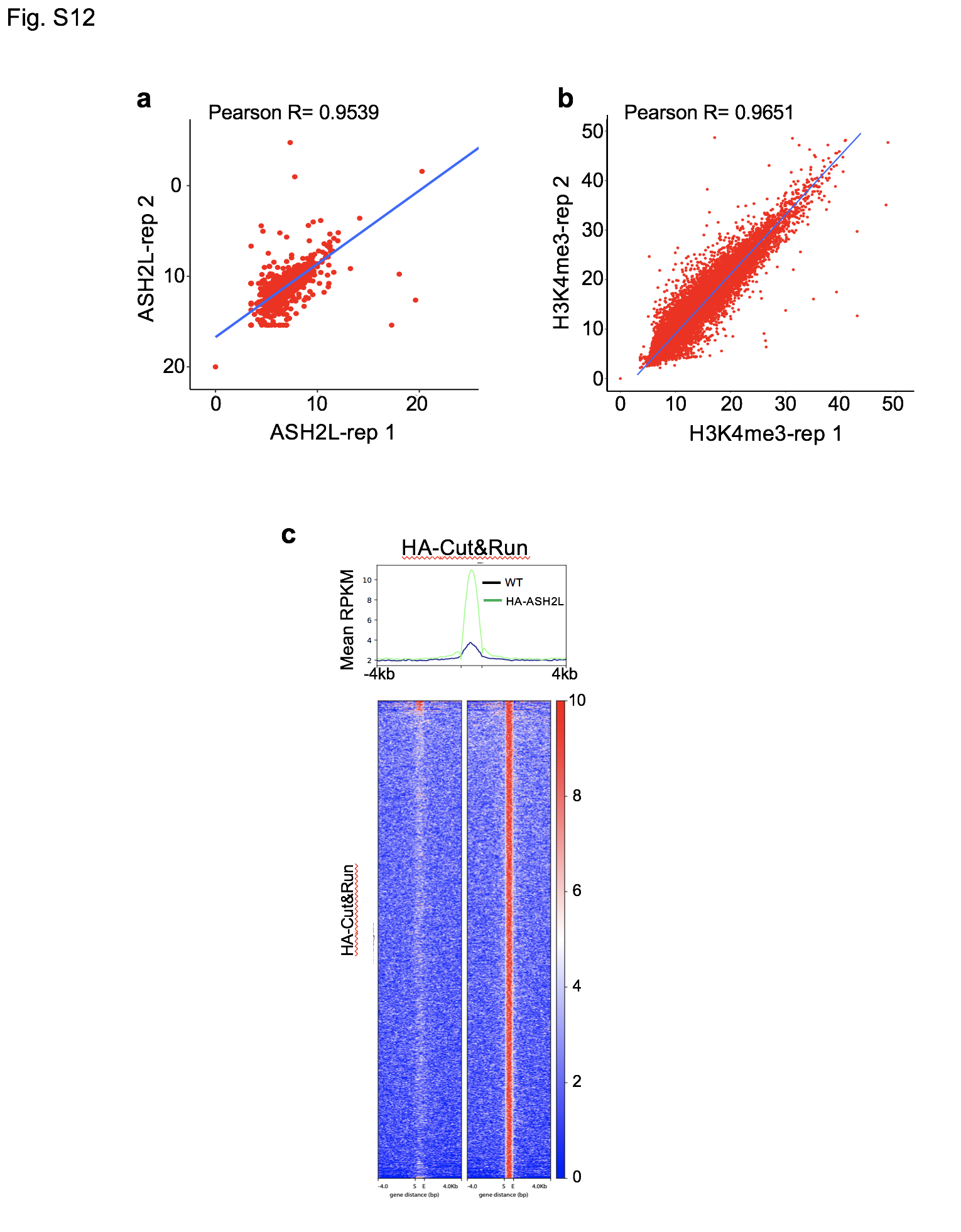
**

**Supplementary Figure 13 | Biological duplicates for ASH2L and H3K4me3 CUT&RUN show good correlation and signal-to-noise ratio.** This figure is related to main Figure 6. **a,b**, Scatter plots for peaks in two independent biological replicates of HA (a) or H3K4me3 (b) CUT&RUN. Pearson correlation coefficient for two samples were shown on top. **c**, Heatmap for HA peaks in the control E14 parental cell line and the HA-ASH2L cell line. Merged signals from biological duplicates were shown, with the heat map key at right.

**SUPPLEMENTARY TABLES**

**Supplementary Table 1.** Methyl Chemical Shift of ASH2L^202-534^ bound to DPY30. This is related to Figure 4.

| **Residue** | **CM1^a^** | **QM1^a^** | **CM2^a^** | **QM2^a^** |  | **Residue** | **CM1^a^** | **QM1^a^** | **CM2^a^** | **QM2^a^** |
| --- | --- | --- | --- | --- | --- | --- | --- | --- | --- | --- |
| L225 | 20.459 | 0.661 | 21.885 | 0.75 |  | V391 | 18.047 | 1.171 | n.d. | n.d. |
| L231 | 20.948 | 0.767 | 22.188 | 0.832 |  | L392 | 19.826 | 0.163 | n.d. | n.d. |
| L238 | 19.794 | 0.947 | 22.092 | 0.966 |  | I396 | 10.303 | -0.874 |  |  |
| I250 | 12.364 | 0.322 |  |  |  | L398 | 23.874 | 0.697 | n.d. | n.d. |
| L251 | 19.437 | 0.207 | 24.297 | 0.489 |  | L408 | n.d. | n.d. | n.d. | n.d. |
| L264 | n.d.^b^ | n.d. | n.d. | n.d. |  | L417 | 21.913 | -0.492 | 22.938 | 0.03 |
| L266 | n.d. | n.d. | n.d. | n.d. |  | I418 | 12.245 | 0.618 |  |  |
| I274 | 10.333 | 0.549 |  |  |  | L424 | 21.157 | 0.297 | 22.659 | 0.324 |
| L278 | 20.459 | 0.636 | 23.211 | 0.845 |  | V432 | 20.154 | 0.877 | n.d. | n.d. |
| L283 | 19.257 | 0.351 | 23.385 | 0.539 |  | L439 | 19.779 | 0.54 | 23.441 | -0.238 |
| V287 | 16.882 | -0.355 | 17.179 | -0.049 |  | I447 | 11.562 | 0.077 |  |  |
| L288 | 19.461 | 1.142 | 24.123 | 0.855 |  | I448 | 10.728 | 0.485 |  |  |
| L289 | n.d. | n.d. | n.d. | n.d. |  | V454 | 17.723 | 0.734 | n.d. | n.d. |
| L291 | 20.615 | 0.654 | 22.66 | 0.844 |  | V458 | 18.456 | 0.79 | 18.659 | 0.612 |
| L298 | n.d. | n.d. | n.d. | n.d. |  | I463 | 10.616 | -0.123 |  |  |
| I300 | 8.308 | 0.564 |  |  |  | V467 | n.d. | n.d. | n.d. | n.d. |
| L305 | 20.588 | 1.238 | 22.974 | 0.938 |  | I472 | 12.475 | 0.57 |  |  |
| V307 | n.d. | n.d. | n.d. | n.d. |  | L474 | n.d. | n.d. | n.d. | n.d. |
| V308 | 16.942 | 0.951 | 17.776 | 0.905 |  | V480 | n.d. | n.d. | n.d. | n.d. |
| V316 | 17.723 | 0.734 | 21.441 | 1.012 |  | I482 | 13.957 | 0.094 |  |  |
| V322 | 18.408 | -0.545 | n.d. | n.d. |  | L495 | 20.52 | 0.316 | 21.173 | 0.069 |
| I331 | 13.053 | 0.645 |  |  |  | V508 | 19.812 | 1.213 | n.d. | n.d. |
| V333 | n.d. | n.d. | n.d. | n.d. |  | V509 | n.d. | n.d. | n.d. | n.d. |
| L344 | 22.929 | 0.373 | 23.052 | 1.032 |  | L513 | n.d. | n.d. | n.d. | n.d. |
| L350 | 19.329 | 0.304 | 23.416 | 0.378 |  | V516 | n.d. | n.d. | n.d. | n.d. |
| L353 | 19.284 | 0.581 | n.d. | n.d. |  | L517 | n.d. | n.d. | n.d. | n.d. |
| L357 | n.d. | n.d. | n.d. | n.d. |  | V520 | n.d. | n.d. | n.d. | n.d. |
| I378 | 9.422 | 0.744 |  |  |  | V524 | 19.023 | 0.822 | n.d. | n.d. |

^a^Methyl resonances are arbitrarily listed without stereospecific assignment for Leu and Val. For Ile, CM1 and QM1 are equivalent to CD1 and QD1, respectively.

^b^Not determined due to strong ambiguity.

**Supplementary Table 2.** Cryo-EM Data Collection, Refinement, and Validation Statistics. This is related to Figure 6, S4 and S5.

|  | **4-MLL1-NCP,**  **Class01**  **(EMD-21542)**  **(PDB: 6W5I)** | **4-MLL1-NCP,**  **Class02**  **(EMD-21543)**  **(PDB: 6W5M)** | **4-MLL1-NCP,**  **Class05**  **(EMD-21544)**  **(PDB: 6W5N)** |
| --- | --- | --- | --- |
| **Data Collection and Processing** | | | |
| Magnification | 29,000 |  | |
| Voltage (kV) | 300 |  |  |
| Electron exposure (e-/Å^2^) | 64 |  |  |
| Defocus range (μm) | -1.5 to -2.5 |  |  |
| Pixel size (Å) | 1.00 |  |  |
| Symmetry imposed | C1 |  |  |
| Initial particle images (no.) | 1,287,711 |  |  |
| Final particle images (no.) | 13,086 | 27,730 | 23,236 |
| Map resolution (Å) | 6.9 | 4.6 | 6.0 |
| FSC threshold | 0.143 | 0.143 | 0.143 |
| **Refinement** | | | |
| Initial model used (PDB code) | 6PWV | 6PWV | 6PWV |
| Model resolution (Å) | 7.6 | 6.5 | 6.9 |
| FSC threshold | 0.5 | 0.5 | 0.5 |
| Map sharpening *B* factor (Å^2^) | -442.70 | -177.74 | -199.18 |
| **Model composition** | | | |
| Non-hydrogen atoms | 19,672 | 19,574 | 19,667 |
| Protein residues | 1,741 | 1,729 | 1,740 |
| Nucleotides | 292 | 292 | 292 |
| Ligands | - | - | - |
| ***B* factor (Å^2^)** | | | |
| Protein | 214.20 | 195.04 | 221.62 |
| Nucleotide | 50.98 | 31.39 | 51.78 |
| Ligand | - | - | - |
| **Rmsds** | | | |
| Bond lengths (Å) | 0.005 | 0.005 | 0.005 |
| Bond angles (°) | 0.674 | 0.673 | 0.671 |
| **Validation** | | | |
| MolProbity score | 2.68 | 2.59 | 2.69 |
| Clashscore | 44.52 | 36.36 | 44.38 |
| Poor rotamers (%) | 1.63 | 1.58 | 1.63 |
| **Ramachandran plot** | | | |
| Favored (%) | 94.21 | 94.06 | 94.09 |
|  |  |  |  |
| Allowed (%) | 5.79 | 5.94 | 5.91 |
| Disallowed (%) | 0 | 0 | 0 |

**Supplementary Table 3.** Survey of IDR content in histone methyltransferases. This is related to Figure 2a.

**
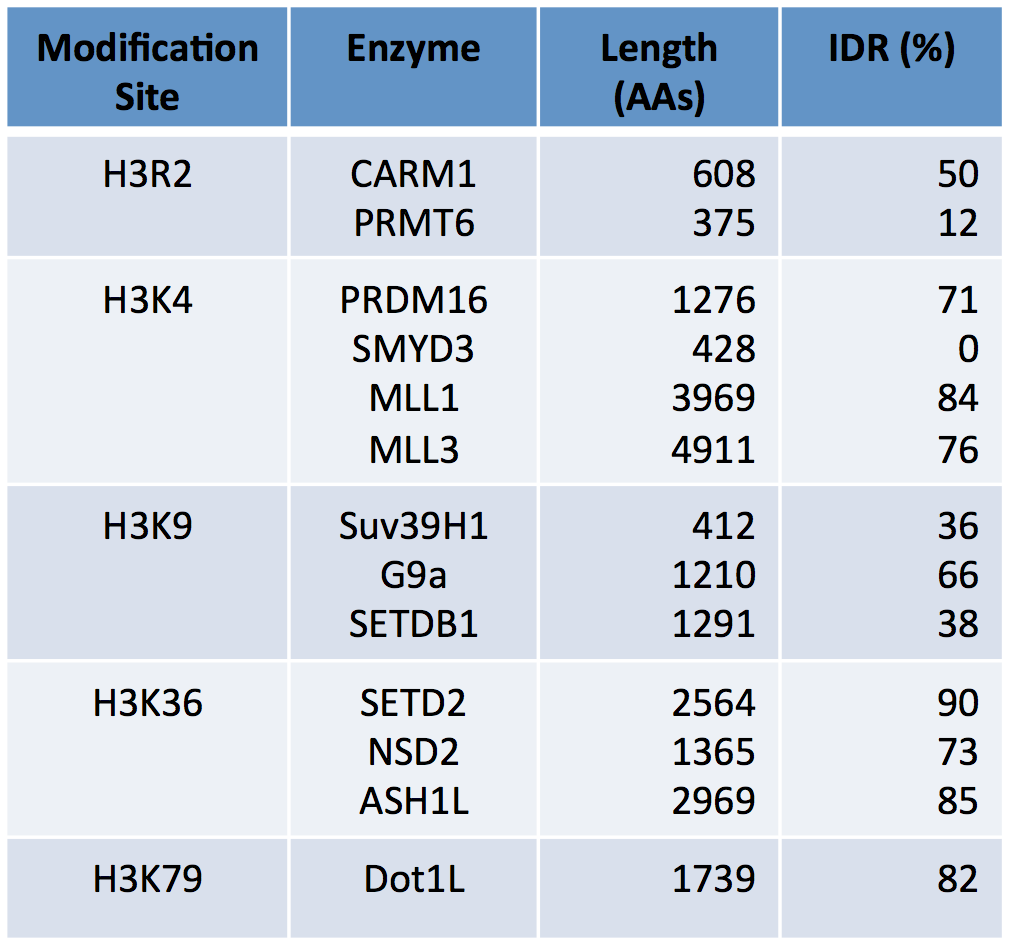
**

**Supplementary Table 4.** gRNA and primer sequence information. This is related to Figure 6c.

**gRNAs:**

| gRNA1-1 sense | CACCGCCCTCTGATCTGTAGCGCAG |
| --- | --- |
| gRNA1-1 antisense | AAACCTGCGCTACAGATCAGAGGGC |
| gRNA1-2 sense | CACCGAGCTGGGTGGTGGACAATGC |
| gRNA1-2 antisense | AAACGCATTGTCCACCACCCAGCTC |
| gRNA1-3 sense | CACCGAAGTGCCCAGGGATGATTGA |
| gRNA1-3 antisense | AAACTCAATCATCCCTGGGCACTTC |
| gRNA2-1 sense | CACCGTCCTGTGAGGTCCTGCGAAA |
| gRNA2-1 antisense | AAACTTTCGCAGGACCTCACAGGAC |
| gRNA2-2 sense | CACCGTGAGGCTAAGGTAATTCAGC |
| gRNA2-2 antisense | AAACGCTGAATTACCTTAGCCTCAC |
| gRNA2-3 sense | CACCGcatctctgcgtatagaccac |
| gRNA2-3 antisense | AAACgtggtctatacgcagagatgC |

**Primers:**

| CHIP-region 1-F | AGGTCTAACTCAGGCTCCCG |
| --- | --- |
| CHIP-region 1-R | ACTGAAGTGACATGTGCGTGTG |
|  |  |
| CHIP-region 2-F | TGCTGCATTGCCTGTCTTGCT |
| CHIP-region 2-R | GGTTGCTTACACCTGCCTGTAAC |

**SUPPLEMENTARY REFERNCES**

1. Schneider, C.A., W.S. Rasband, and K.W. Eliceiri, *NIH Image to ImageJ: 25 years of image analysis.* Nat Methods, 2012. **9**(7): p. 671-5.

2. Kucukelbir, A., F.J. Sigworth, and H.D. Tagare, *Quantifying the local resolution of cryo-EM density maps.* Nature Methods, 2014. **11**(1): p. 63-65.

3. Afonine, P.V., et al., *New tools for the analysis and validation of cryo-EM maps and atomic models.* Acta Crystallogr D Struct Biol, 2018. **74**(Pt 9): p. 814-840.

4. Rubin, A.J., et al., *Coupled Single-Cell CRISPR Screening and Epigenomic Profiling Reveals Causal Gene Regulatory Networks.* Cell, 2019. **176**(1-2): p. 361-376 e17.
