# Supplementary material for: Mechanism for DPY30 and ASH2L intrinsically disordered regions to modulate the MLL/SET1 activity on chromatin
Source: Nat Commun. 2021 May 19;12:2953. doi: 10.1038/s41467-021-23268-9 (PMC8134635; doi:10.1038/s41467-021-23268-9)
Supplement: Supplementary file 2 — Reporting Summary [file 41467_2021_23268_MOESM2_ESM.pdf]

nature research

Corresponding author(s): Yali Dou

Last updated by author(s): Mar 24, 2021

Reporting Summary

Nature Research wishes to improve the reproducibility of the work that we publish. This form provides structure for consistency and transparency in reporting. For further information on Nature Research policies, see our Editorial Policies and the Editorial Policy Checklist.

Statistics

For all statistical analyses, confirm that the following items are present in the figure legend, table legend, main text, or Methods section.

n/a	Confirmed	
☐	☒	The exact sample size (*n*) for each experimental group/condition, given as a discrete number and unit of measurement
☐	☒	A statement on whether measurements were taken from distinct samples or whether the same sample was measured repeatedly
☐	☒	The statistical test(s) used AND whether they are one- or two-sided<br>*Only common tests should be described solely by name; describe more complex techniques in the Methods section.*
☒	☐	A description of all covariates tested
☒	☐	A description of any assumptions or corrections, such as tests of normality and adjustment for multiple comparisons
☐	☒	A full description of the statistical parameters including central tendency (e.g. means) or other basic estimates (e.g. regression coefficient) AND variation (e.g. standard deviation) or associated estimates of uncertainty (e.g. confidence intervals)
☐	☒	For null hypothesis testing, the test statistic (e.g. *F*, *t*, *r*) with confidence intervals, effect sizes, degrees of freedom and *P* value noted<br>*Give P values as exact values whenever suitable.*
☒	☐	For Bayesian analysis, information on the choice of priors and Markov chain Monte Carlo settings
☒	☐	For hierarchical and complex designs, identification of the appropriate level for tests and full reporting of outcomes
☐	☒	Estimates of effect sizes (e.g. Cohen's *d*, Pearson's *r*), indicating how they were calculated

Our web collection on statistics for biologists contains articles on many of the points above.

Software and code

Policy information about availability of computer code

Data collection	IconNMR; NMRPipe; NMRFAM-Sparky; PRIMUS (GUI version);
Data analysis	C-I-TASSER; LOMETS; FG-MD; MotionCorr2; CTFFIND4.1; cryoSPARC; PHENIX; MolProbity; Bowtie2 (v2-2.2.4); SAMtools (v1.5); deepTools3 (v3.2.1); BEDTools; MACS (v 1.4.2); GraphPad Prism 7.0; ImageJ.

For manuscripts utilizing custom algorithms or software that are central to the research but not yet described in published literature, software must be made available to editors and reviewers. We strongly encourage code deposition in a community repository (e.g. GitHub). See the Nature Research guidelines for submitting code & software for further information.

Data

Policy information about availability of data

All manuscripts must include a data availability statement. This statement should provide the following information, where applicable:

- Accession codes, unique identifiers, or web links for publicly available datasets
- A list of figures that have associated raw data
- A description of any restrictions on data availability

ChIP-seq dataset for DPY30 and MLL1 were downloaded from GEO GSE26136 and GEO GSE107406, respectively. Dataset for HA-ASH2L and H3K4me3 is accessible with accession code GSE146933 [https://www.ncbi.nlm.nih.gov/geo/query/acc.cgi?acc=GSE146933].
Cryo-EM structures for the 5MLL1-NCP complex and the ySET1-NCP can be accessed by PDB ID: 6PWV [https://www.rcsb.org/structure/6PWV] and EMDB: EMD-20512; PDB: 6KIX [https://www.rcsb.org/structure/6KIX], EMDB: EMD-0694; and PDB: 6UGM [https://www.rcsb.org/structure/6UGM], EMDB: EMD- 20765.
The accession numbers for the 4-MLL1-NCP class01; class02; and class05 cryo-EM structures, reported in this study, are PDB 6W5I [https://www.rcsb.org/structure/6W5I] and EMDB EMD-21542 ; PDB 6W5M: [https://www.rcsb.org/structure/6W5M] and EMDB: EMD-21543; PDB 6W5N: [https://www.rcsb.org/structure/6W5N] and EMDB: EMD-21544, respectively.

Source data are provided with this paper.

Field-specific reporting

Please select the one below that is the best fit for your research. If you are not sure, read the appropriate sections before making your selection.

☒ Life sciences ☐ Behavioural & social sciences ☐ Ecological, evolutionary & environmental sciences

For a reference copy of the document with all sections, see nature.com/documents/nr-reporting-summary-flat.pdf

Life sciences study design

All studies must disclose on these points even when the disclosure is negative.

Sample size	We used biological duplicates for the CUT&RUN experiments and biological triplicates for the CHIP-qPCR experiments. The sample size selection was based on statistical considerations.
Data exclusions	There is no data exclusion in our study.
Replication	All experiments have been successfully replicated for at least three times. Reproducibility statement is included in Methods section.
Randomization	Randomization was not considered since all experiments were performed in vitro.
Blinding	No blinding was used since all experiments were performed in vitro by one person and it is not possible to set up blinding experiments.

Reporting for specific materials, systems and methods

We require information from authors about some types of materials, experimental systems and methods used in many studies. Here, indicate whether each material, system or method listed is relevant to your study. If you are not sure if a list item applies to your research, read the appropriate section before selecting a response.

Materials & experimental systems

n/a	Involved in the study	
☐	☒	Antibodies
☐	☒	Eukaryotic cell lines
☒	☐	Palaeontology and archaeology
☒	☐	Animals and other organisms
☒	☐	Human research participants
☒	☐	Clinical data
☒	☐	Dual use research of concern

Methods

n/a	Involved in the study	
☒	☐	ChIP-seq
☒	☐	Flow cytometry
☒	☐	MRI-based neuroimaging

Antibodies

Antibodies used	Rabbit anti-H3K4mel (Abeam, cat ab8895, 1:20000), Rabbit anti-H3K4me2 (Millipore, cat# 07-030, 1:20000), Rabbit-anti H3K4me3 (Millipore, cat# 07-473, 1:10000), Rabbit anti-Histone H3 (Abeam, #ab1791, 1:20000), anti-Rabbit lgG Horseradish Peroxidaselinked whole antibody (GE Healthcare, #NA934, 1:10000), and anti-HA (clone C29F4) rabbit monoclonal antibody (Cell signaling technology, cat #3724, 1:1000).
Validation	The primary antibodies against modified histone H3K4 are validated by the manufacture using modified H3 peptides and further confirmed in the lab for specificity using histone H3 with or without MLL1 mediated methylation. The primary antibody for HA is validated, by manufacture by immunoblot and immunofluorecence for the antigen.

Eukaryotic cell lines

Policy information about cell lines

Cell line source(s)	E14tg2a (E14) (ATCC, #CRL-1821TM)
Authentication	Authentication was done by karyotyping
Mycoplasma contamination	All cell lines are tested negative for mycolasma contamination using the Lookout® Mycoplasma PCR Detection Kit (SIGMA ALDRICH, #MP0035) according to the manufacturer's instructions

Commonly misidentified lines<br>(See ICLAC register)	No commonly misidentified lines were used in this study
